# Functional Consequences for Apoptosis by Transcription Elongation Regulator 1 (TCERG1)-Mediated *Bcl-x* and *Fas/CD95* Alternative Splicing

**DOI:** 10.1371/journal.pone.0139812

**Published:** 2015-10-13

**Authors:** Marta Montes, Mayte Coiras, Soraya Becerra, Cristina Moreno-Castro, Elena Mateos, Jara Majuelos, F. Javier Oliver, Cristina Hernández-Munain, José Alcamí, Carlos Suñé

**Affiliations:** 1 Department of Molecular Biology, Instituto de Parasitología y Biomedicina “López Neyra”, Consejo Superior de Investigaciones Científicas (IPBLN-CSIC), PTS, Granada, Spain; 2 AIDS Immunopathology Unit, Centro Nacional de Microbiología, Instituto de Salud Carlos III, Majadahonda, Madrid, Spain; 3 Department of Cell Biology and Immunology, Instituto de Parasitología y Biomedicina “López Neyra”, Consejo Superior de Investigaciones Científicas (IPBLN-CSIC), PTS, Granada, Spain; IISER-TVM, INDIA

## Abstract

Here, we present evidence for a specific role of the splicing-related factor TCERG1 in regulating apoptosis in live cells by modulating the alternative splicing of the apoptotic genes *Bcl-x* and *Fas*. We show that TCERG1 modulates *Bcl-x* alternative splicing during apoptosis and its activity in *Bcl-x* alternative splicing correlates with the induction of apoptosis, as determined by assessing dead cells, sub-G1-phase cells, annexin-V binding, cell viability, and cleavage of caspase-3 and PARP-1. Furthermore, the effect of TCERG1 on apoptosis involved changes in mitochondrial membrane permeabilization. We also found that depletion of TCERG1 reduces the expression of the activated form of the pro-apoptotic mitochondrial membrane protein Bak, which remains inactive by heterodimerizing with Bcl-x_L_, preventing the initial step of cytochrome *c* release in Bak-mediated mitochondrial apoptosis. In addition, we provide evidence that TCERG1 also participates in the death receptor-mediated apoptosis pathway. Interestingly, TCERG1 also modulates *Fas/CD95* alternative splicing. We propose that TCERG1 sensitizes a cell to apoptotic agents, thus promoting apoptosis by regulating the alternative splicing of both the *Bcl-x* and *Fas/CD95* genes. Our findings may provide a new link between the control of alternative splicing and the molecular events leading to apoptosis.

## Introduction

It is estimated that more than 90% of multiexonic human genes undergo alternative splicing, resulting in a widespread tool to achieve proteomic diversity [1, 2]. Alternative splicing plays an important role in gene expression regulation that underlies numerous physiological processes, such as neuronal signaling, stress responses, and apoptosis [3–5]. Changes in the *cis*- or *trans*-regulation of this process can cause multiple pathologies as a result of general or specific aberrant pre-mRNA processing, underscoring the fundamental importance of this regulatory process [6].

Apoptosis is one form of programmed cell death required for proper development and homeostasis in multicellular organisms [7, 8]. Dysregulation of this process could also result in pathological alterations, such as neurodegenerative disorders, autoimmune diseases, or cancer [9–11]. There are two main apoptosis pathways: the intrinsic, or mitochondrial, pathway and the extrinsic, or death receptor, pathway. A large number of genes involved in both pathways are regulated by alternative splicing, allowing the production of distinct protein isoforms, which often differ in function, from one common messenger RNA precursor (pre-mRNA). For example, the death receptor Fas/CD95, which encodes a transmembrane death receptor, can be alternatively processed, excluding exon 6 from the mRNA transcript to produce a soluble isoform that inhibits apoptosis [12, 13]. Several genes of the Bcl-2 family are also regulated via alternative splicing to generate proteins with opposite functions in cell death. The equilibrium between anti-apoptotic and pro-apoptotic isoforms is essential for death or survival decisions [14].

The best characterized example of this equilibrium is *Bcl-x*, which belongs to the Bcl-2 family and is alternatively spliced to produce anti-apoptotic long (Bcl-x_L_) and pro-apoptotic short (Bcl-x_S_) isoforms [15]. Bcl-x_L_ is widely expressed and present at high levels in oncogenic tissues. Overexpression of Bcl-x_L_ confers resistance to apoptotic stimuli and chemotherapeutic agents, favoring metastasis [15, 16]. Bcl-x_S_ is primarily expressed in lymph nodes, thymus, ovary, and other tissues that require an elevated rate of cell replacement [17]. Bcl-x_S_ induces cell death and sensitizes several cell types to apoptotic agents, although whether this protein plays a direct or indirect role in this process remains controversial [18–21]. Bcl-x_L_ plays a key role in apoptosis by preventing cytochrome *c* release from the mitochondria into the cytosol through a number of diverse protein-protein interactions [22]. However, the mechanism by which Bcl-2 proteins provoke apoptosis is still under debate [23]. Consistent with a potential model for this mechanism, the pro-apoptotic Bax and Bak proteins remain blocked in healthy cells by anti-apoptotic proteins, such as Bcl-x_L_ [24]. Upon apoptotic induction, other Bcl2 family members disrupt these interactions, thereby displacing Bax and/or Bak from Bcl-x_L_ and other anti-apoptotic proteins, allowing them to be activated by self-oligomerization. In this model, the ratio between Bcl-x_L_ and Bcl-x_S_ isoforms is important to maintain the critical interactions that can lead to cell health or death. The mechanism by which the ratio between both Bcl-x isoforms is regulated, resulting in the expression of the Bcl-x_L_ isoform, which prevents Bax and/or Bak from activating apoptosis, remains unknown.

The two Bcl-x isoforms are generated from two alternative 5’ splice sites (ss) located in exon 2 of the pre-mRNA. Several *cis*-elements present in *Bcl-x* pre-mRNA and RNA-binding proteins recognizing these elements regulate the alternative splicing of *Bcl-x* [25–29]. The physiological relevance of these interactions that lead to specific changes in the alternative splicing of *Bcl-x* has been demonstrated in several studies. Staurosporine, a general kinase inhibitor and inducer of the intrinsic pathway of apoptosis, switches the production of Bcl-x toward the Bcl-x_S_ isoform by interfering with the protein kinase C (PKC) signaling pathway through a 361-nucleotide region (SB1) on the *Bcl-x* pre-mRNA that is located upstream of the *Bcl-x 5’* ss [28]. Similarly, ceramide and protein phosphatase-1, which are regulators of apoptosis, modulate the use of *Bcl-x* 5’ ss by dephosphorylating members of the SR family of splicing proteins [30, 31]. Because the fine-tuned balance between Bcl-x_L_ and Bcl-x_S_ is important for cell survival or death, modulation of *Bcl-x* alternative splicing is considered useful for new therapeutic developments in apoptosis-related diseases [32–34].

Recently, we showed that the elongation and splicing-related factor TCERG1 regulates the alternative splicing of *Bcl-x* by modulating the rate of RNA polymerase II (RNAPII) transcription [35]. These results together with previous reports implicating TCERG1 in the regulation of apoptosis [36, 37] suggest a role for TCERG1 in the regulation of cell death. TCERG1 is a nuclear protein that contains multiple protein domains, notably the three WW domains in the amino-terminus and the six FF repeat domains in the carboxyl-terminus [38]. TCERG1 associates with hyperphosphorylated RNAPII and transcriptional elongation and splicing components through both the WW and FF domains [37, 39–41]. Given these and other functional data showing the effects of TCERG1 on the alternative splicing of reporter minigene constructs [42–44], TCERG1 has been proposed as a potential factor in coordinating transcriptional elongation and splicing. Consistent with this hypothesis, we recently demonstrated that TCERG1 increases the rate of RNAPII transcription of *Bcl-x in vivo* to promote the splicing of the pro-apoptotic Bcl-x_S_ isoform via the SB1 regulatory element in exon 2 of *Bcl-x* [35]. This result underscores the importance of the functional coupling between transcription and alternative splicing in the regulation of gene expression, particularly for *Bcl-x* [45]. Given these data, it was of interest to investigate whether the effect of TCERG1 on the alternative splicing of *Bcl-x* has functional consequences for apoptosis. Here, we investigate the role of TCERG1 in apoptosis and report that TCERG1 affects both the intrinsic and extrinsic apoptosis pathways. We propose that TCERG1 sensitizes the cell to apoptotic agents, thereby promoting apoptosis by regulating the alternative splicing of the apoptotic genes *Bcl-x* and *Fas/CD95*.

## Materials and Methods

### Cell cultures and treatments

HEK293T and HeLa cells (American Type Culture Collection) were grown in Dulbecco’s modified Eagle Medium (DMEM, Gibco) containing 10% fetal bovine serum (FBS) (Gibco) supplemented with penicillin (100 U)/streptomycin (0.1 mg/ml) (Lonza,), and 4 mM L-glutamine (Gibco). The GFP (control) and TCERG1-knocked down T-Rex-HEK293T cell lines have been described previously [46]. The cells were maintained in high-glucose DMEM containing 10% FBS supplemented with penicillin/streptomycin and L-glutamine. We used 200 μg/ml hygromycin (Invitrogen) for selection, 5 μg/ml tetracycline (Sigma) for shRNA induction, and 15 μg/ml blasticidin (Invitrogen) for the inhibition of tetracycline induction. Jurkat cells stably transfected with pGeneClip-shTCERG1-C1 (Jurkat shTCERG1-C1) or Jurkat shTCERG1-(1-3-4) have been described previously [46]. The cells were grown in RPMI 1640 medium (BioWhittaker) containing 10% fetal calf serum (PAN Biotech GmbH) and supplemented with 2 mM L-glutamine, 100 μg/ml streptomycin, 100 U/ml penicillin, and 5 μg/ml puromycin (Invitrogen) at 37°C. For induction of the intrinsic apoptosis pathway, staurosporine (Sigma) was used at 1 μM for 12 h in T-Rex-HEK293T cells and 0.5 μM for 1 h in Jurkat cells, unless otherwise indicated.

Peripheral blood mononuclear cells (PBMCs) were isolated from blood of healthy donors by centrifugation through a Ficoll-Hypaque gradient (GE Healthcare, Milwaukee, WI) and cultured in the same conditions as Jurkat cells. PBMCs were activated by treatment with 5μg/ml PHA (Sigma-Aldrich, St. Louis, MO) and 300 units/ml IL-2 (Chiron, Emeryville, CA).

### Plasmids

The pEFBOST7-TCERG1 plasmid has been described previously [41, 47]. pcDNA3-FLAG-Bcl-x_S_ was generated by subcloning the cDNA of the Bcl-x_S_ isoform into the pcDNA3-FLAG vector by standard PCR using the following oligonucleotides: BclEcoRI-F, 5’-GGGGAATTCTCATTTCCGACTGAAG-3’, and BclXbaI-R, 5’-GGGGAATTCTCATTTCCGACTGAAG-3’.

### Transfection assays

Jurkat cells were transfected by electroporation with an EasyJet Plus Electroporator (Equibio). In brief, 5 x 10^6^ cells were collected in 350 μl of RPMI without supplementation and mixed with 5 μg of plasmid DNA in an electroporation cuvette with a 4 mm electrode gap (Equibio). The cells were transfected by one pulse at 280 V and 1500 μF. After transfection, the cells were incubated in supplemented RPMI for 48 h at 37°C. Then, the cells were treated as indicated and processed for analysis. In each transfection, the plasmid pCDNA3-GFP was included to evaluate the transfection efficiency, which was approximately 60–75%.

Resting PBMCs were transiently transfected with the Easyjet Plus Electroporator. In brief, 10^7^ PBMCs were collected in 350μl of RPMI without supplement and mixed with 1μg/10^6^ cells of plasmid DNA. Cells were transfected in a cuvette with 4mm electrode gap, at 320V, 1500μF and maximum resistance. After transfection, cells were incubated in supplemental RPMI for 18h, at 37°C. For flow cytometry, an in-house developed monoclonal antibody against TCERG1 and a secondary antibody conjugated to Alexa 633 (Molecular Probes, Eugene, OR) were used. pEYFP-C1 vector was used as control of transfection.

### RNA extraction and reverse transcription (RT)-PCR analysis

Total RNA was extracted from cells with peqGOLD TriFast (peQlab) according to the manufacturer’s protocol. The RNA was digested with 10 U of RNase-free DNase (Roche) for 30 min at 37°C. After DNase treatment at 70°C for 5 min, 1 μg of endogenous RNA or 400 ng of plasmid-derived RNA was reverse-transcribed using oligo(dT)_15_ and Moloney murine leukemia virus reverse transcriptase (Invitrogen) for 1 h at 37°C. One-tenth of the resulting cDNA was amplified by PCR using the following oligonucleotides: X3, 5’-ATGGCAGCAGTAAAGCAAGCG-3’, and X2, 5’-TCATTTCCGACTGAAGAGTGA-3’, for endogenous *Bcl-x* detection; Fend-F, 5’-GCACCAAATGTGAACATGG-3’, and Fend-R, 5’-CCATTCTTTCGAACAAAGCC-3’, for endogenous *Fas* isoforms. After 30 cycles at 60°C, the products were separated on a 2% agarose gel and the intensity of the bands was quantified using Quantity One software (Bio-Rad). The quantification of the transcripts isoform of Fas/CD95 was performed by real-time PCR using the iTaq Universal SYBR Green Supermix and the iCycler thermal station (Bio-Rad) with the following oligonucleotides: FasE6, 5’-TAACTTGGGGTGGCTTTGTC-3’, and FasE7, 5’-TCCTTTCTGTGCTTTCTGCAT-3’, for the long pro-apoptotic isoform; and FasE5E7, 5’-CAAAGAGGAAGTGAAGAGAAA-3’ and FasE7Rev, 5’-AGGATTTAAAGTTGGAGATTCA-3’, for the short anti-apoptotic isoform. Glyceraldehyde-3-phosphate dehydrogenase (GAPDH) was used as an internal gene control and amplified with the oligonucleotides GAPDH-F, 5’-ATGGGGAAGGTGAAGGTCG-3’, and GAPDH-R, 5’- GGGTCATTGATGGCAACAATATC-3’. The values are represented as (*E* Fas)^ΔCT Fas^/(*E* GAPDH)^ΔCT GAPDH^, where *E* is the PCR efficiency and Δ*CT* = (the cycle threshold [*CT*] for control cells)—(*CT* for TCERG1 knockdown).

### Western blot analysis

A fraction of the cells were lysed in cold T7 buffer (20 mM HEPES [pH 7.9], 150 mM NaCl, 5 mM EDTA, 1% NP-40, 1 mM dithiothreitol, protease inhibitor mixture [Complete, Roche], and 1 mM PMSF). The proteins were separated by 10% sodium dodecyl sulfate-polyacrylamide gel electrophoresis (SDS-PAGE), transferred to a nitrocellulose membrane (Amersham Biosciences), and then incubated with specific antibodies against TCERG1 [41] at 1:2000 and CDK9 (sc-484, Santa Cruz Biotechnology) at 1:1000. Peroxidase-conjugated secondary antibodies (PerkinElmer Life Science) were used at a 1:5000 dilution, and the bound antibodies were detected by enhanced chemiluminescence (PerkinElmer Life Science). The apoptosis proteins were separated by 12.5% SDS-PAGE, transferred to nitrocellulose membranes, and detected using antibodies against caspase-3 (sc-7148), cleaved Parp-1 (sc-56196), Bcl-x_L_ (sc-23958), and Bcl-2 (sc-7382) (Santa Cruz Biotechnology) at 1:500 dilution and β-actin (A5441, Sigma) at 1:5000. A densitometric analysis was performed using ImageJ software. The gel bands were quantified, and the background noise was subtracted from the images. The relative ratio of optical density units was calculated relative to the gel band corresponding to the internal control (β-actin) for each lane and each type of sample. The p20+p17/procaspase-3 ratio was calculated to determine the values of the caspase isoforms.

### Cell cycle

Approximately 1 x 10^6^ Jurkat cells were collected in polystyrene tubes and washed twice with phosphate-buffered saline (PBS). The cells were fixed with 70% ethanol at 4°C for at least 15 min. After washing with PBS, the cells were diluted in a solution of PBS containing 100 μg/ml RNase (Roche) and 40 μg/ml propidium iodide (PI, Sigma). After incubating for 20 minutes at 37°C, the samples were analyzed using a FACSCalibur flow cytometer (BD Biosciences, Clontech). The cell cycle data were analyzed using the FlowJo v7.6.5 software.

### Annexin-V binding assays

Approximately 1 x 10^6^ cells were stained with annexin-V conjugated to fluorescein isothiocyanate (FITC) or phycoerythrin (Immunostep S.L.); fluorescence was measured by flow cytometry. The data were analyzed using CellQuest software.

### Active caspase-3 and caspase-8 measurement

The quantification of active caspase-3 and caspase-8 was performed using the Caspase-Glo 3/7 and the Caspase-Glo 8 assays (Promega) according to the manufacturer’s instructions. The luminescent signal (relative light units, RLUs), which was directly proportional to caspase activation, was measured using an Infinite F200 TECAN Luminometer. The data were normalized to the protein concentration measured by the Bradford assay.

### Cell viability

Cell viability was determined using the CellTiter-Glo® Luminescent Cell Viability Assay (Promega), following the manufacturer’s instructions. Briefly, 1 x 10^5^ cells were harvested by centrifugation, washed twice with 1X PBS, and diluted in lysis buffer. After incubation for 10 min at room temperature to stabilize the luminescent signal, the cell lysates were deposited in an opaque-walled multiwell plate and analyzed using an Infinite F200 TECAN Luminometer. The data were normalized to the protein concentration measured by the Bradford assay.

### Mitochondrial membrane potential gradient (ΔΨ_m_)

ΔΨ_m_ was measured using the Mitochondrial Staining JC-1 Assay Kit (Sigma) according to the manufacturer’s instructions. Briefly, 1.5 x 10^6^ Jurkat cells were incubated with JC-1 (5,5’,6,6’-tetrachloro-1,1’,3,3’-tetraethylbenzimidazolylcarbocyanine iodide) staining solution, and both green and red fluorescence were measured in the FL1 and FL2 channels, respectively, by flow cytometry. Mitochondrial depolarization was calculated by measuring the green fluorescence intensity.

### Analysis of intracellular Bak expression

Bak-specific immunofluorescence was analyzed after treatment with 1μM staurosporine for 3 h. Cells were fixed in 1% paraformaldehyde (PFA) for 1 h. After washing with 1X PBS, the cells were permeabilized with 1% Tween-20 in 1X PBS and then incubated with a Bak primary antibody (AM03, Calbiochem) and a secondary antibody conjugated to FITC. The analysis was performed using a FACSCalibur flow cytometer (BD Biosciences Clontech) with CellQuest software.

### Mitochondrial cytochrome c release

The release of cytochrome *c* from the mitochondrial intermembrane space into the cytosol was measured using InnoCyte Flow Cytometric Cytochrome *c* Release Kit (Calbiochem). Briefly, 5 x 10^6^ cells were treated or not with 1μM staurosporine for 3 h, permeabilized, and then fixed with 1% PFA in 1X PBS. After staining with a monoclonal antibody against cytochrome *c* obtained from Calbiochem (Merck Chemicals Ltd., Nottingham, UK) and a secondary antibody conjugated to FITC, cytochrome *c* release was measured by flow cytometry. The release of cytochrome *c* into the cytosol was also analyzed by fluorescence microscopy. Living cells treated or not with staurosporine were adhered to PolyPrep slides (Sigma-Aldrich) and fixed with 2% PFA. Immunofluorescence assays were then performed as previously described [48] using the antibody against cytochrome *c*. Images were obtained with a Leica DMI 4000B Inverted Microscope (Leica Microsystems, Barcelona, Spain).

### Statistical analysis

The statistical analysis was performed using GraphPad Prism 5.0 software (GraphPad Software Inc., San Diego, CA). Two-tailed Student’s *t*-tests and Mann-Whitney *U*-test analysis were used to compare means between the samples and their respective controls. The *p* values are represented by asterisks (*, *p* < 0.05; **, *p* < 0.01). The absence of an asterisk indicates that the change relative to the control was not statistically significant.

## Results

### TCERG1 modifies the *Bcl-x* splicing pattern upon staurosporine-mediated apoptosis induction

**Fig 1:**
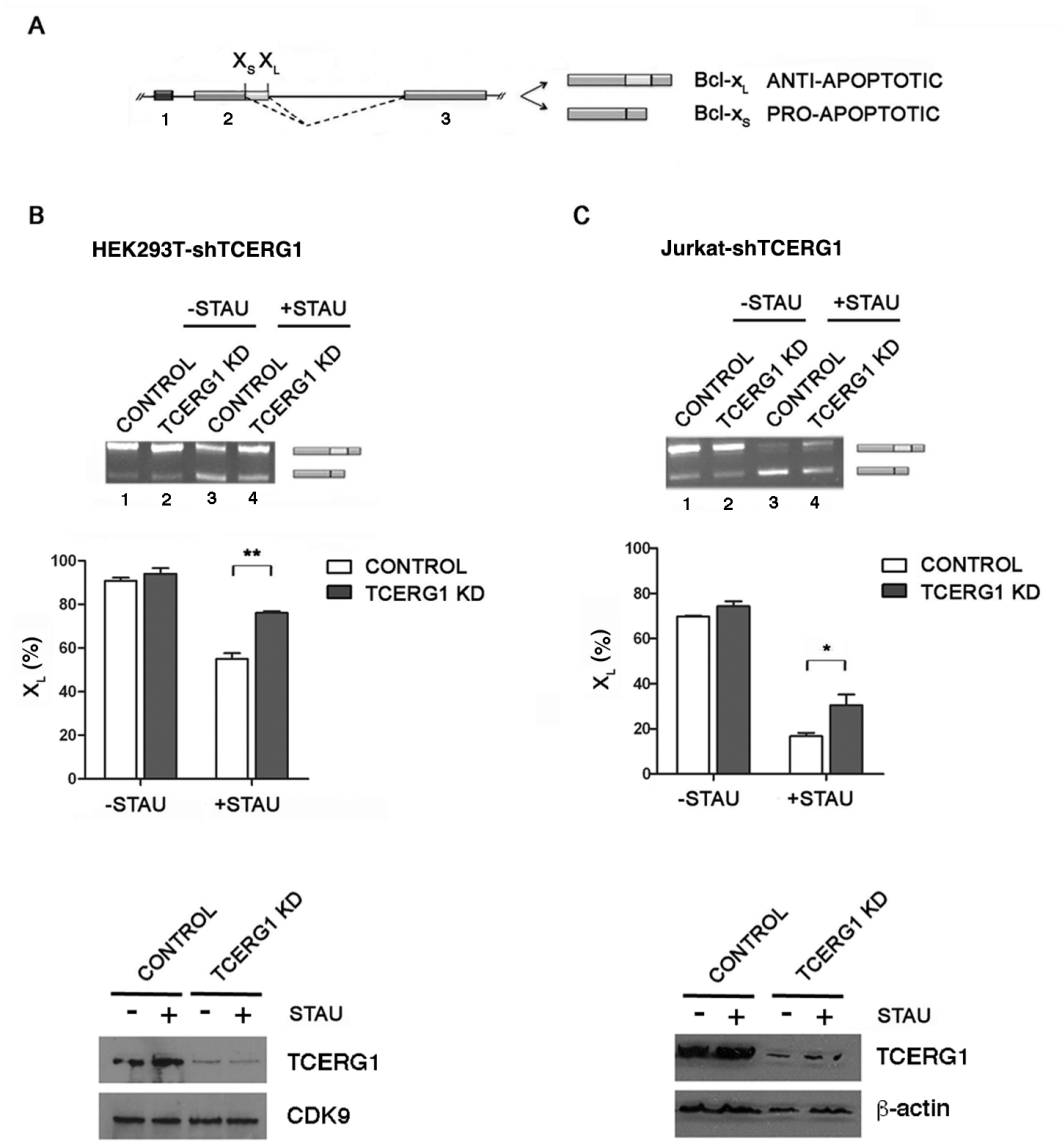
Regulation of *Bcl-x* alternative splicing by TCERG1 upon staurosporine-induced apoptosis. (A) Schematic representation of the structure of the *Bcl-x* gene, with exons (boxes) and introns (lines). Two splice variants derived from the *Bcl-x* gene, anti-apoptotic Bcl-x_L_ and pro-apoptotic Bcl-x_S_, are generated via alternative 5’ splice site (X_S_ and X_L_) selection within exon 2. The dotted lines indicate the alternative splicing events. (B and C) Knockdown of TCERG1 (KD) increased the level of the anti-apoptotic Bcl-x_L_ isoform after staurosporine treatment. Control (lanes 1 and 3) and TCERG1 knockdown T-Rex-HEK293T cells (lanes 2 and 4) (B) and Jurkat cells (C) were treated with 1 μM staurosporine for 12 h and 0.5 μM staurosporine for 1 h, respectively. After total RNA extraction, the RNA splicing variants were amplified by RT-PCR, and the products were separated on 2% agarose gels. The graphs show the densitometric results as the percentage of the Bcl-x_L_ isoform in three independent experiments (means ± SEM). *, *p* < 0.05, **, *p* < 0.01. A fraction of the cell lysates was analyzed by immunoblotting for TCERG1, CDK9, and ß-actin.

We conducted *in vivo* assays to determine whether TCERG1 influences apoptosis. First, we analyzed the effect of TCERG1 knockdown on *Bcl-x* alternative splicing during apoptosis. Bcl-x proteins are involved in the mitochondrial pathway of apoptosis; therefore, we used staurosporine, a protein kinase inhibitor that mainly induces the mitochondrial death pathway. We analyzed the splicing pattern of *Bcl-x* after 12 h of 1 μM staurosporine treatment by RT-PCR in T-Rex-HEK293T cells, in which the expression of control shRNA or shRNA targeting TCERG1 was induced by the addition of tetracycline. In control cells, we observed an increase of Bcl-x_S_ and a decrease of Bcl-x_L_ (Fig 1B, lanes 1 and 3), as reported previously [28]. This pattern was partially reversed upon TCERG1 knockdown (Fig 1B, lanes 2 and 4). To perform this protocol under optimal conditions, we used Jurkat cells, which have been extensively used to study apoptotic gene modulation. We used stable TCERG1-knocked down Jurkat cells to analyze the splicing pattern of *Bcl-x* upon staurosporine treatment by RT-PCR. Similar to previous results, addition of staurosporine produced the Bcl-x_S_ isoform almost exclusively (Fig 1C, lanes 1 and 3). We observed a shift in the *Bcl-x* splicing pattern toward the Bcl-x_L_ isoform upon TCERG1 depletion more clearly evident in the staurosporine-treated Jurkat cells (Fig 1C, lanes 2 and 4). The analysis of cell lysates showed that the T-Rex-HEK293T and Jurkat cells with TCERG1 knockdown expressed significantly lower amounts of TCERG1 protein than the control cells (Fig 1B and 1C, bottom). Taken together, these data indicate that TCERG1 modulates *Bcl-x* alternative splicing during apoptosis.

### TCERG1 depletion decreases the proportion of apoptotic cells induced by staurosporine

**Fig 2:**
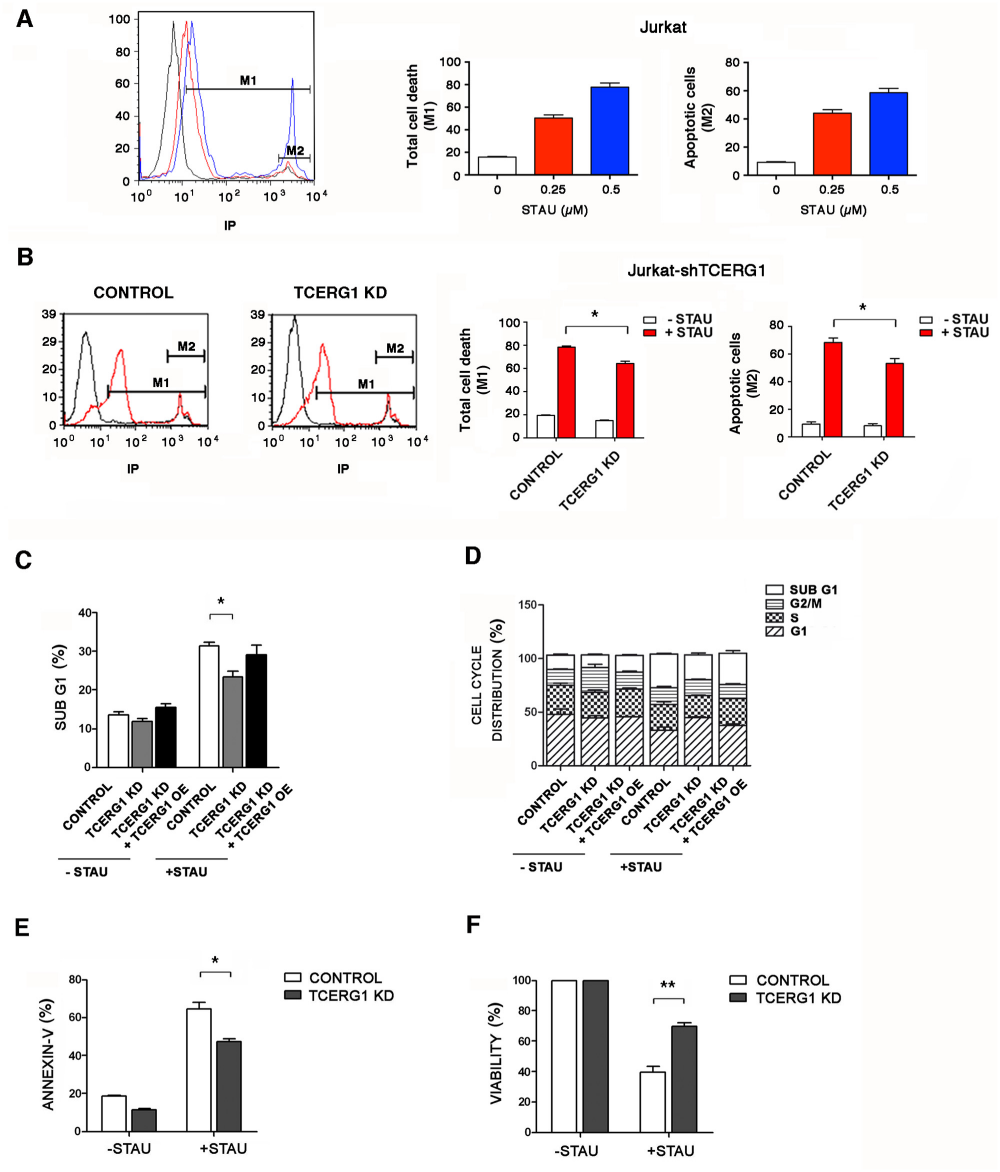
Effect of TCERG1 knockdown on the staurosporine-induced apoptotic pathway. (A) Effect of staurosporine treatment on Jurkat cells. Flow cytometry analysis of Jurkat cells stained with PI without (black) or with the addition of 0.25 (red) or 0.5 μM staurosporine (blue) for 1 h. M1: marker line representing the percentage of dead cells. M2: marker line indicating the population of necrotic cells. The histogram of flow cytometry and bar graphs of the percentage of M1 and M1-M2 cells are shown. The graphs show the data from three independent experiments (means ± SEM). (B) Depletion of TCERG1 decreases the percentage of total cell death. Jurkat cells with control or stable TCERG1 mRNA interference (TCERG1 KD) were stained with PI without (black) or with the addition of 0.5 μM staurosporine for 1 h (red). The histogram of flow cytometry and bar graphs of the percentage of M1 and M1-M2 cells are shown. The graph shows the data from three independent experiments (means ± SEM). *, *p* < 0.05. (C) Depletion of TCERG1 decreases the percentage of the sub-G1 cell population. Jurkat cells were transiently transfected with control or the siTCERG1 alone (TCERG1 KD) or with a TCERG1 expression plasmid (TCERG1 KD + TCERG1 OE). The graph shows the percentage of cells in the sub-G1 phase of the cell cycle in the absence (-STAU) or presence (+STAU) of 0.5 μM staurosporine for 1 h. Data are from four independent experiments (means ± SEM). *, *p* < 0.05. (D) Cell cycle distribution of stably transfected Jurkat cells. Jurkat cells with control or stable TCERG1 mRNA interference (TCERG1 KD) were transiently transfected with an empty vector or, in the case of TCERG1 KD, with a TCERG1 expression vector (TCERG1 OE). After 48 h, the cells were incubated in the absence (-STAU) or presence of staurosporine (+STAU) at 0.5 μM for 1 h. The cells were fixed and incubated with PI. The cell cycle stages were analyzed by flow cytometry. The graph shows the percentage of cells in each phase of the cell cycle. Data are from three independent experiments (means ± SEM). *, *p* < 0.05. (E) TCERG1 knockdown reduces annexin-V binding after staurosporine treatment. Control and TCERG1 knockdown (KD) Jurkat cells were incubated with annexin-V and analyzed by flow cytometry in the absence (-STAU) or in the presence of 0.5 μM staurosporine (+STAU) for 1 h. The bar graph shows the percentage of annexin-V-positive cells from three independent experiments (means ± SEM). *, *p* < 0.05. (F) TCERG1 knockdown increases the viability of staurosporine-treated cells. Control and TCERG1-knockdown Jurkat cells were incubated without (-STAU) or with 0.5 μM staurosporine (+STAU) for 1 h, and viability was measured by chemiluminescence. The bar graph represents the percentage of viable cells (means ± SEM). Data are from four independent experiments. **, *p* < 0.01.

As a first approach to investigate the relevant biological consequences for the regulation of *Bcl-x* alternative splicing by TCERG1, we measured the percentage of dead Jurkat-shTCERG1 cells upon staurosporine treatment using propidium iodide (PI) (Fig 2A and 2B). Because PI does not enter unfixed live cells, it is commonly used to detect dead cells in a given population. However, this technique does not distinguish between apoptotic and necrotic cells. To better quantify the apoptotic cells, we discarded the cell population with a higher amount of PI, which is thought to constitute the necrotic cells (Fig 2A and 2B, marker M2), from the calculation of total cell death (Fig 2A and 2B, marker M1). We examined the sensitivity of Jurkat cells to different concentrations of staurosporine and observed a dose-dependent effect (Fig 2A). We then analyzed the effect of this treatment on the Jurkat-shTCERG1 cells in the presence of 0.5 mM staurosporine. After 1 h of staurosporine treatment, the cells were analyzed by flow cytometry to evaluate PI staining as a measure of cell death. We found that TCERG1 knockdown reduced the number of apoptotic cells (Fig 2B). To corroborate these data, we performed cell cycle experiments to determine the number of cells in the sub-G1 phase and reveal the discontinuous fragmentation of nuclear DNA during apoptosis, corresponding to apoptotic cells. In the absence of staurosporine treatment, there was no significant difference in the percentage of cells in the sub-G1 population between control and TCERG1-knocked down cells (Fig 2C, -STAU). After apoptotic induction, an increase in sub-G1 phase formation was observed in both Jurkat cell lines. Interestingly, the percentage of cells in the sub-G1 population was significantly lower in TCERG1-knocked down cells compared with control cells (Fig 2C, +STAU). Moreover, transient overexpression of TCERG1 in TCERG1-knocked down cells almost completely recovered the percentage of cells in sub-G1 phase upon staurosporine induction to the level observed in control cells, indicating that the effect observed in the decrease of sub-G1 cells was mainly due to the TCERG1 depletion (Fig 2C). The other phases of the cell cycle were not significantly altered (Fig 2D). To lend additional support to our data, we also performed annexin-V binding assays. Annexin-V specifically binds phosphatidylserine when this molecule is located at the cell surface under apoptotic conditions. After staurosporine induction, we measured the number of annexin-V-positive cells by flow cytometry in our Jurkat cell lines. In agreement with the above data, we observed significantly reduced annexin-positive cells upon TCERG1 knockdown (Fig 2E). Finally, we performed experiments to determine the number of viable cells based on the quantification of the ATP present as an indicator of metabolically active cells. We observed that the knockdown of TCERG1 partially recovered the decrease in cell viability in response to staurosporine (Fig 2F). These findings indicate that TCERG1 depletion reverses the effect of staurosporine-induced apoptosis.

**Fig 3:**
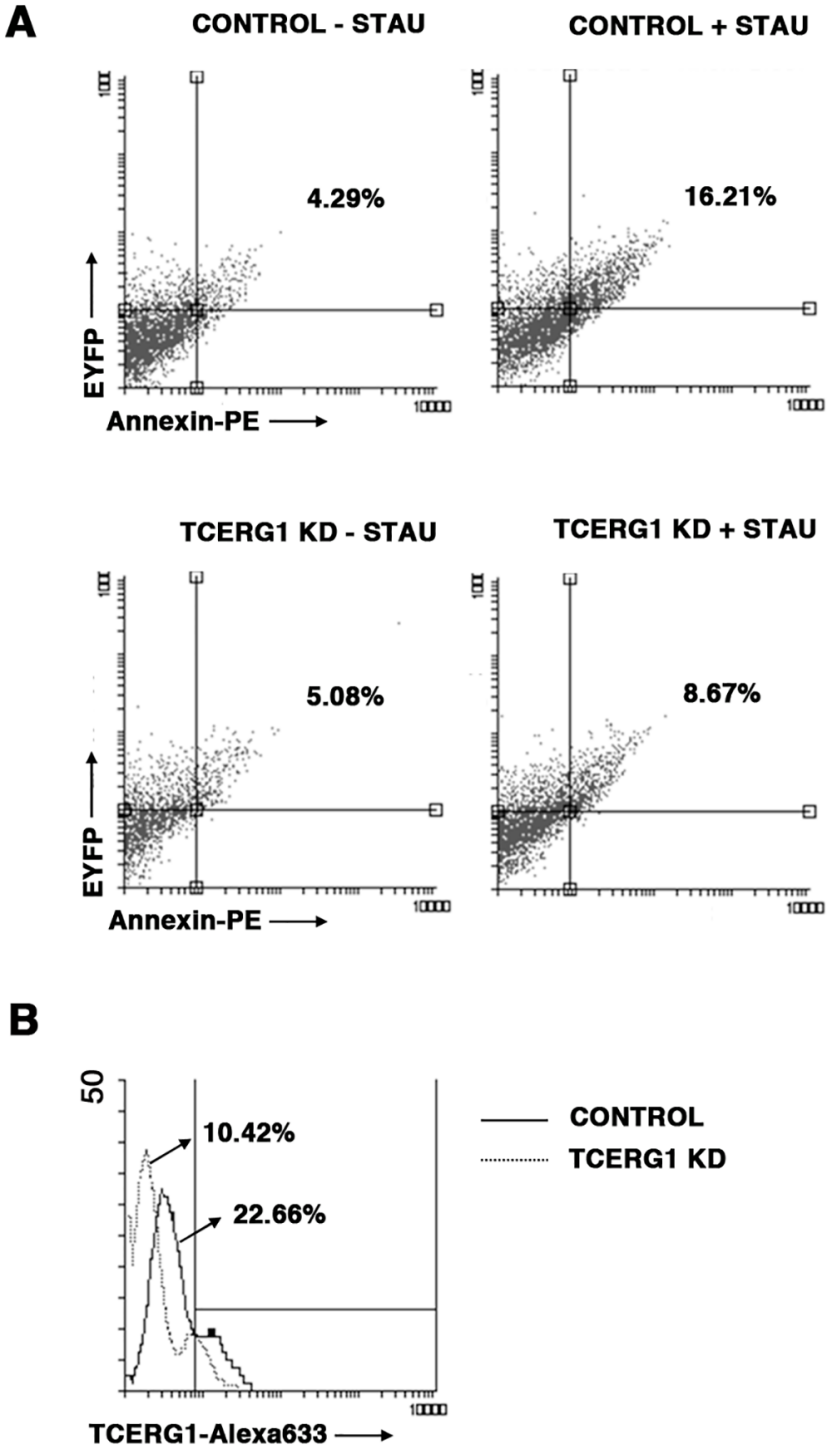
Effect of TCERG1 on apoptosis using PBMCs. (A) Transiently reduced expression of TCERG1 in PBMCs decreases apoptosis induced by staurosporine. Apoptosis induced by staurosporine was measured by flow cytometry in EYFP+ PBMCs transiently transfected with pGeneClip-shTCERG1-(1-3-4) or control vectors after staining with annexin-V-PE. We performed the experiment three independent times. (B) Level of TCERG1 expression was analyzed by flow cytometry.

To demonstrate the physiological relevance of TCERG1 in apoptosis in non-transformed cells, we performed experiments using resting PBMCs isolated from healthy donors. PBMCs were transfected with pGeneClip-shTCERG1-(1-3-4) vectors (rate 1:1:1) or the negative control pGeneClip-shTCERG1-C1, and the pEYFP vector was co-transfected as control of transfection efficiency. Cells were incubated for 3 days with PHA/IL-2 and then split and treated or not with staurosporine 10 μM for 3 h. Early apoptosis was measured in EYFP+ cells by flow cytometry after staining with annexin V. Apoptosis induced by staurosporine was reduced 1.8-fold in TCERG1 knockdown cells, compared to control cells (Fig 3A). The level of expression of TCERG1 was analyzed by flow cytometry using a specific monoclonal antibody and a secondary antibody conjugated to Alexa 633. TCERG1 expression was reduced approximately 2-fold in TCERG1 knockdown cells (Fig 3B).

### Reduction of apoptosis by TCERG1 depletion is associated with a decrease in active caspase-3

**Fig 4:**
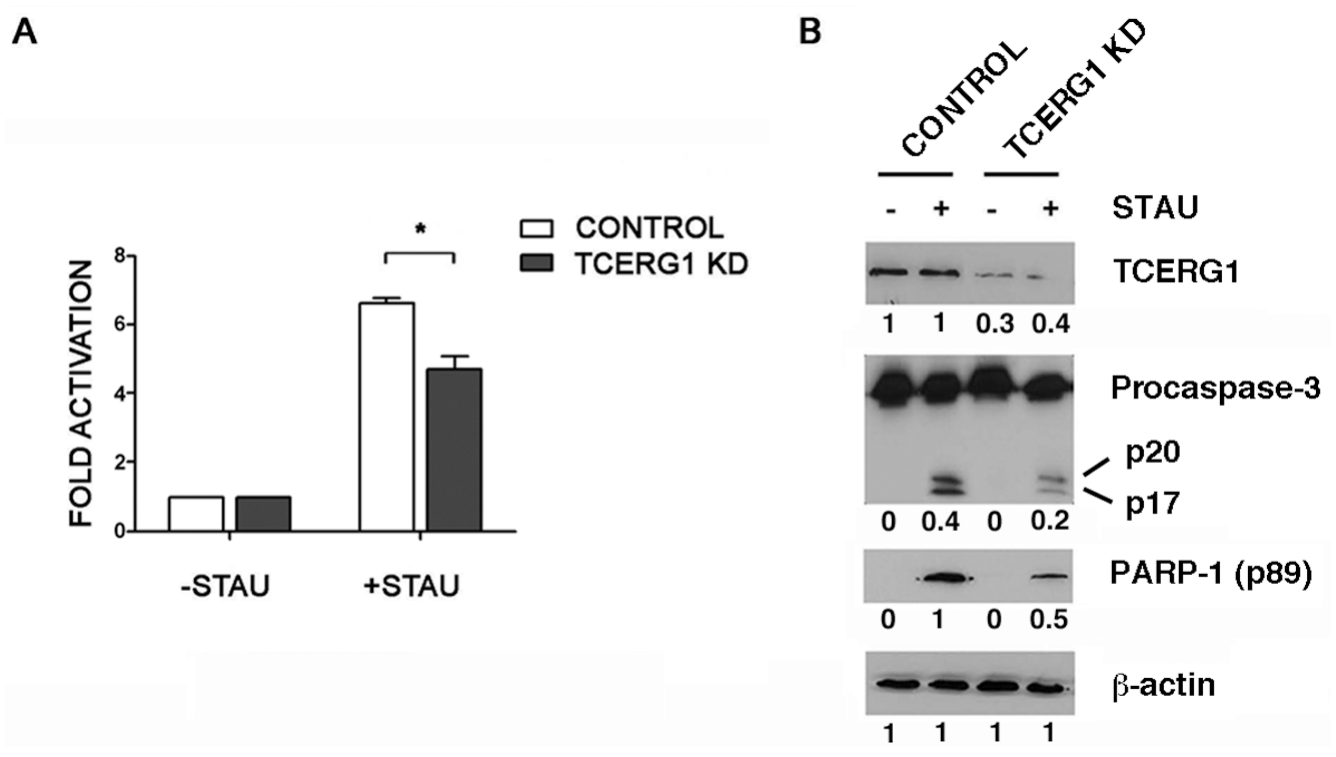
Analysis of caspase-3 activation in TCERG1-depleted cells after staurosporine induction. (A) Activation of caspase-3 was decreased in TCERG1-knockdown Jurkat cells treated with staurosporine. Caspase-3 activation was measured by chemiluminescence in control and TCERG1-knockdown Jurkat cells treated (+STAU) or not with 0.5 μM staurosporine (-STAU) for 1 h. The bar diagram shows the fold activation from four independent experiments (means ± SEM). Data obtained from cells without staurosporine were set as 1. *, *p* < 0.05. (B) Analysis of TCERG1, procaspase-3 cleavage (precursor p35 and cleaved fragments p20/p17), and PARP-1 cleavage (p89) levels. Protein expression was analyzed by immunoblotting using specific antibodies in protein extracts obtained from control and TCERG1-knockdown Jurkat cells treated (+STAU) or not with 0.5 μM staurosporine (-STAU) for 1 h. ß-actin was used as a loading control. The quantification of the bands is shown below each panel.

Caspase-3 activity is currently used as a marker of apoptosis. To measure active caspase-3 in the cells, we used an assay based on the addition of a luciferase substrate that is cleaved in the presence of active caspase-3, leading to a luminescent signal. The number of luciferase units directly correlates with the amount of active caspase-3 present in the cell. We treated the cells with staurosporine and observed a more than 6-fold increase in the quantity of active caspase-3 in control cells, whereas in the TCERG1 knockdown cells, this increase was significantly lower (Fig 4A). Caspase-3 is expressed as a 35 kDa inactive precursor from which the 17 kDa (p17) and 11 kDa (p11) subunits are proteolytically generated during apoptosis. The caspase-3 precursor is first cleaved to generate a p11 subunit and a p20 peptide, which is subsequently cleaved to produce the mature p17 subunit. The active caspase-3 enzyme is a hetero-tetramer composed of two p17 peptides and two p11 subunits. Using specific antibodies against caspase-3, we observed significantly less p20 and p17 upon TCERG1 knockdown (Fig 4B, compare lane 2 to lane 4). The cleavage of the caspase-3 target protein PARP-1 was also analyzed as a marker of caspase activity. PARP-1 is cleaved during apoptosis leading to the appearance of an 89 kDa product. Using an antibody specific for the cleaved protein, we observed that PARP-1 cleavage was reduced upon TCERG1 knockdown (Fig 4B, compare lane 2 to lane 4). These findings confirm that in cells treated with staurosporine, the inhibition of TCERG1 causes a decrease in caspase-3 activation.

### TCERG1 promotes changes in mitochondrial membrane permeabilization and in the emergence of the active form of Bak upon apoptosis induction

**Fig 5:**
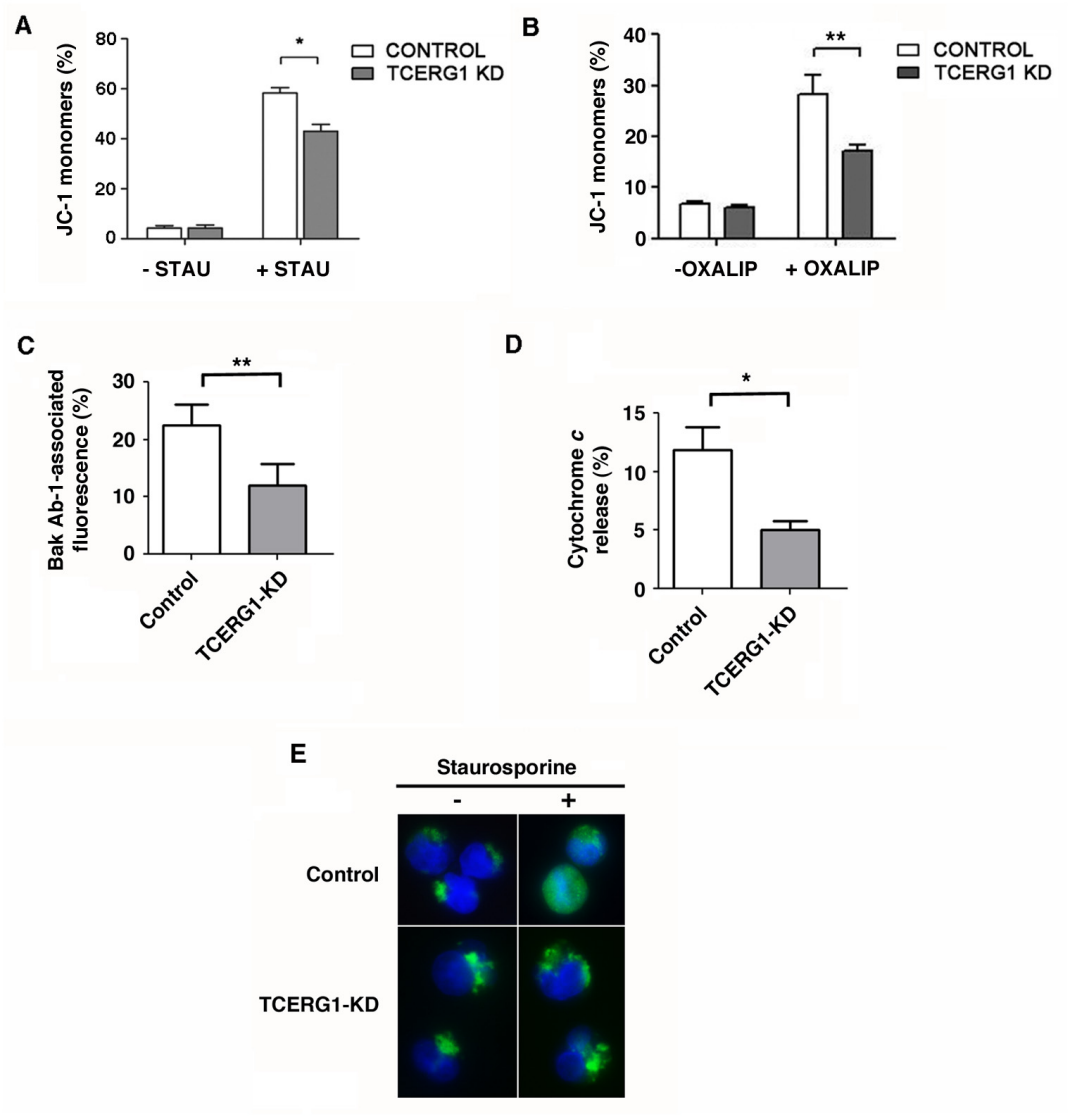
Effect of TCERG1 on staurosporine-mediated apoptosis involves changes in mitochondrial membrane permeabilization and in the emergence of the active form of Bak. (A) TCERG1 knockdown decreases the mitochondrial membrane potential dissipation caused by staurosporine. Control and TCERG1-knockdown (KD) Jurkat cells were incubated without (-STAU) or with 0.5 μM staurosporine (+STAU) for 1 h and stained with JC-1 dye. Green fluorescence was measured by flow cytometry, and the data were analyzed using FlowJo software. The bar diagram shows the quantification of JC-1 monomers from two independent experiments (means ± SEM). *, *p* < 0.05. (B) TCERG1 mRNA interference decreases the mitochondrial membrane potential dissipation caused by oxaliplatin in Jurkat cells. Control and shTCERG1 Jurkat cells were incubated without (-OXALIP) or with 20 μM oxaliplatin (+OXALIP) for 18 h and stained with JC-1 dye. Green fluorescence was measured by flow cytometry, and the data were analyzed using CellQuest and GraphPad software. The bar diagram shows the quantification of JC-1 monomers from two independent experiments (means ± SEM). **, p < 0.01. (C) Changes in Bak-associated fluorescence were quantified by flow cytometry after intracellular staining with the Ab-1 antibody. (D and E) The release of cytochrome *c* was analyzed by flow cytometry and immunofluorescence. The graphs show data from three independent experiments (means ± SEM). *, *p* < 0.05, **, *p* < 0.01.

During apoptosis, a cascade of key events occurs in the mitochondria, including the dissipation of the mitochondrial membrane potential gradient (ΔΨm). ΔΨm is a parameter of mitochondrial function and therefore a suitable indicator of cell death. To monitor mitochondrial health, we used the membrane-permeable JC-1 dye. In healthy cells, the dye is incorporated into the mitochondria, spontaneously forming red-fluorescent JC-1 aggregates. In apoptotic cells, the dissipation of the gradient favors the diffusion of the dye, resulting in green-fluorescent JC-1 monomers. We measured the fluorescence emission in control and TCERG1-depleted cells by flow cytometry. We observed a loss of green monomer fluorescence upon TCERG1 knockdown in staurosporine-treated cells, indicating a higher ΔΨm (Fig 5A). This finding suggests that TCERG1 affects the mitochondrial membrane potential in the early stages of programmed cell death caused by staurosporine treatment.

To test wether other apoptotic inducers are able to elicit a similar response as that observed for staurosporine, we used doxorubicin and oxaliplatin. Doxorubicin induces DNA damage through topoisomerase II inhibition and free radical generation via a redox reaction [49], and the DNA damage-inducer oxaliplatin favors the pro-apoptotic Bcl-x_S_ variant [50]. We did not observe differences in the percentage of dead Jurkat-shTCERG1 and control cells upon doxorubicin treatment using PI, whereas we did find that TCERG1 knockdown reduced the percentage of dead cells upon oxaliplatin treatment (data not shown). Moreover, we observed a loss of green monomer fluorescence upon TCERG1 knockdown in oxaliplatin-treated cells, indicating a higher ΔΨm (Fig 5B). Interestingly, the signaling routes elicited by staurosporine and oxaliplatin appear to converge into the same splicing regulatory event controlling the production of the pro-apoptotic Bcl-x_S_ [51] (see Discussion).

The Bcl-2 family member Bak is an integral mitochondrial membrane protein that exposes an amino-terminal-occluded epitope upon apoptosis induction with staurosporine or other stimuli [52]. This conformational change converts Bak into a primed state and provokes the formation of higher-order Bak homo-oligomers that are thought to be responsible for the mitochondrial dysfunctions that characterize the commitment of a cell to apoptosis, such as cytochrome *c* release. Bak binds to Bcl-x_L_, and this heterodimerization may prevent the cells from undergoing apoptotic death [52–56]. To investigate the mechanisms underlying the effects of TCERG1 on apoptosis, we measured the change in Bak-associated immunofluorescence by flow cytometry using an antibody specific for the amino-terminal-occluded epitope of Bak [52]. Interestingly, we observed reduced appearance of Bak amino-terminal-associated immunofluorescence in the shTCERG1 cells (Fig 5C), indicating that the cells were less perturbed or damaged upon TCERG1 depletion. The release of cytochrome *c* from Jurkat cells upon staurosporine treatment was also analyzed by flow cytometry (Fig 5D) and immunofluorescence (Fig 5E). The results showed a decrease in cytochrome *c* release in shTCERG1 cells than control cells. Together, these data show that upon staurosporine induction, TCERG1 knockdown reduces the availability of the amino-terminus of Bak and prevents the initial step of cytochrome *c* release under Bak-mediated mitochondrial apoptosis.

### The effect of TCERG1 on staurosporine-mediated apoptosis involves changes in the Bcl-x_L_/Bcl-x_S_ ratio

**Fig 6:**
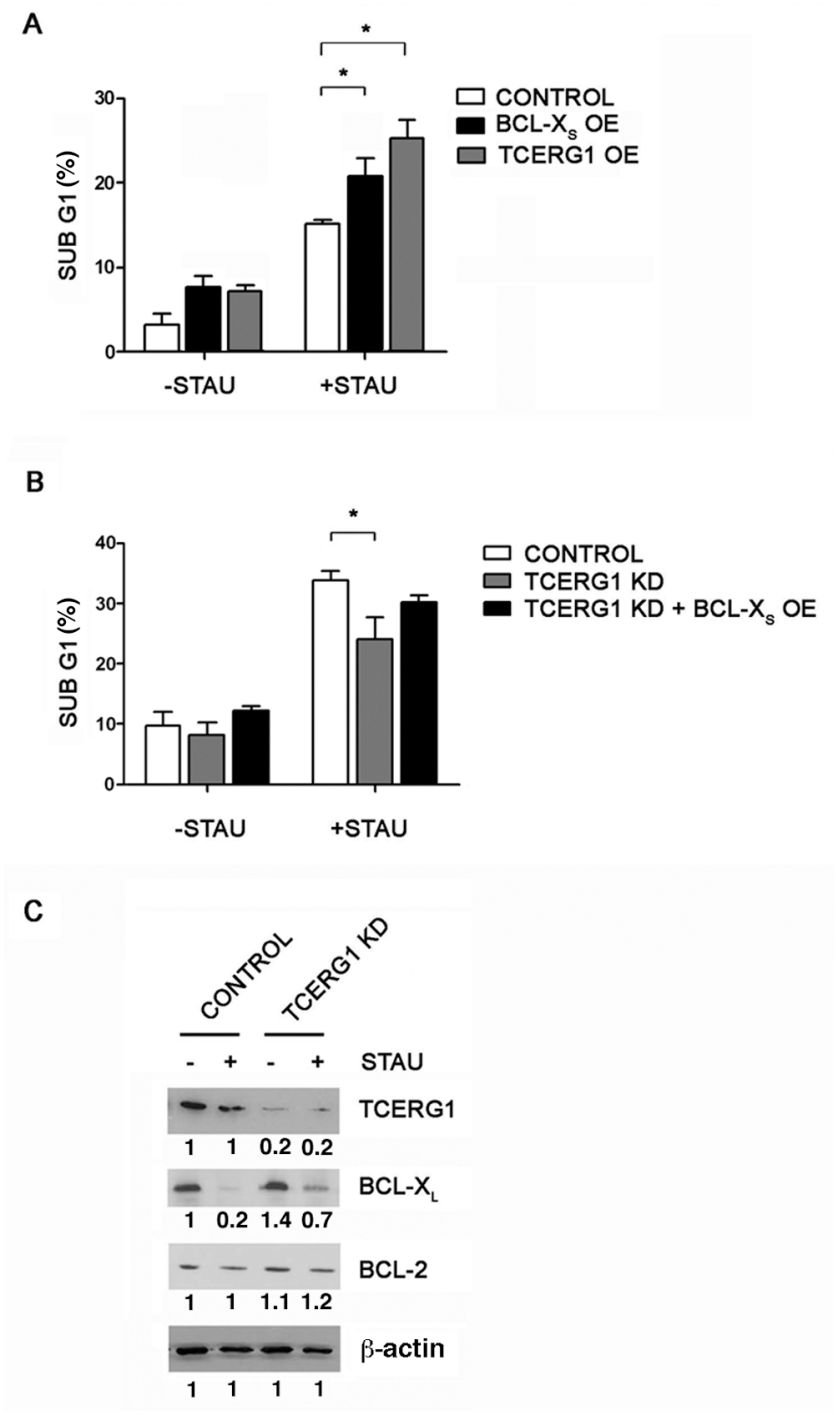
TCERG1’s effect on staurosporine-mediated apoptosis is caused by a change in the Bcl-x_L_/Bcl-x_S_ ratio. (A) Overexpression of Bcl-x_S_ and TCERG1 favors staurosporine-induced apoptosis. Jurkat cells were transiently transfected with an empty vector (control) or with Bcl-x_S_ or TCERG1 expression plasmids (Bcl-x_S_ OE and TCERG1 OE, respectively). Cells were treated with (+STAU) or without 0.025 μM staurosporine (-STAU) for 1 h, and the cell cycle was then analyzed. The graph shows the percentage of cells in sub-G1 phase from three independent experiments (means ± SEM). *, *p* < 0.05. (B) The transient overexpression of Bcl-x_S_ in TCERG1-knockdown cells increases the number of apoptotic cells. Control and TCERG1-knockdown Jurkat cells were transiently transfected with an empty vector or a Bcl-x_S_ expression plasmid (TCERG1 KD + Bcl-x_S_ OE) in the case of TCERG1-knockdown cells. The cells were treated with (+STAU) or without 0.5 μM staurosporine (-STAU) for 1 h, and the cell cycle was then analyzed. The graph represents the percentage of cells in sub-G1 phase, measured by flow cytometry, from three independent experiments (means ± SEM). *, *p* < 0.05. (C) Depletion of TCERG1 decreases the level of Bcl-x_L_ protein in cells upon staurosporine treatment. Cell extracts from control and TCERG1 knockdown (KD) Jurkat cells with (+STAU) or without treatment with 0.5 μM staurosporine (-STAU) for 1 h were analyzed by immunoblotting with specific antibodies against TCERG1, Bcl-x, Bcl-2, and β-actin. The quantification of the bands is shown below each panel.

As Bcl-x plays a role in maintaining the mitochondrial membrane potential [57], we determined whether the effect of TCERG1 on ΔΨm is caused by a change in the level of Bcl-x_L_ and Bcl-x_S_ isoforms through alternative splicing regulation. The Bcl-x_L_ isoform produces an anti-apoptotic protein that prevents mitochondrial apoptosis [58]. However, although the Bcl-x_S_ protein favors apoptosis, it is not clear whether this protein is sufficient to directly antagonize the anti-apoptotic effects of Bcl-2 and Bcl-x_L_ [15] or whether it plays a secondary role in sensitizing cells to pro-apoptotic agents [18, 19]. To determine whether Bcl-x_S_ favors apoptosis in Jurkat cells, we transiently transfected a plasmid expressing this isoform and analyzed the cell cycle by flow cytometry. We observed a slight increase in the sub-G1 cell population upon Bcl-x_S_ overexpression in the absence of staurosporine, and this effect was statistically significant after staurosporine treatment (Fig 6A). Similarly, the overexpression of TCERG1 increased the percentage of the sub-G1 cell population in the presence of staurosporine (Fig 6A). An interpretation of these results is that the increase in the Bcl-x_S_ isoform elicited by TCERG1 overexpression promoted apoptosis in response to staurosporine treatment. Consistent with this hypothesis, the overexpression of Bcl-x_S_ under TCERG1 depletion partially reversed the decrease in apoptotic cells observed after apoptosis induction in the absence of TCERG1 (Fig 6B), suggesting that the effect observed after the knockdown of TCERG1 was partly due to the reduction of the Bcl-x_S_ isoform.

We next analyzed the protein expression levels using specific antibodies. We observed a decrease in Bcl-x_L_ in the control cells after staurosporine treatment (Fig 6C, lanes 1 and 2) that was partially recovered in the TCERG1-knocked down cells (Fig 6C, lanes 3 and 4), supporting the functional results for alternative splicing and apoptosis. Unfortunately, we could not detect Bcl-x_S_ with commercially available antibodies (data not shown). To lend specificity to these results, we did not observe any difference in Bcl-2 expression between the control and TCERG1-knocked down cells (Fig 6C). Together, these results suggest that TCERG1 depletion counteracts staurosporine-induced apoptosis by favoring the Bcl-x_L_ over the Bcl-x_S_ isoform.

### TCERG1 depletion decreases death receptor-mediated apoptosis

**Fig 7:**
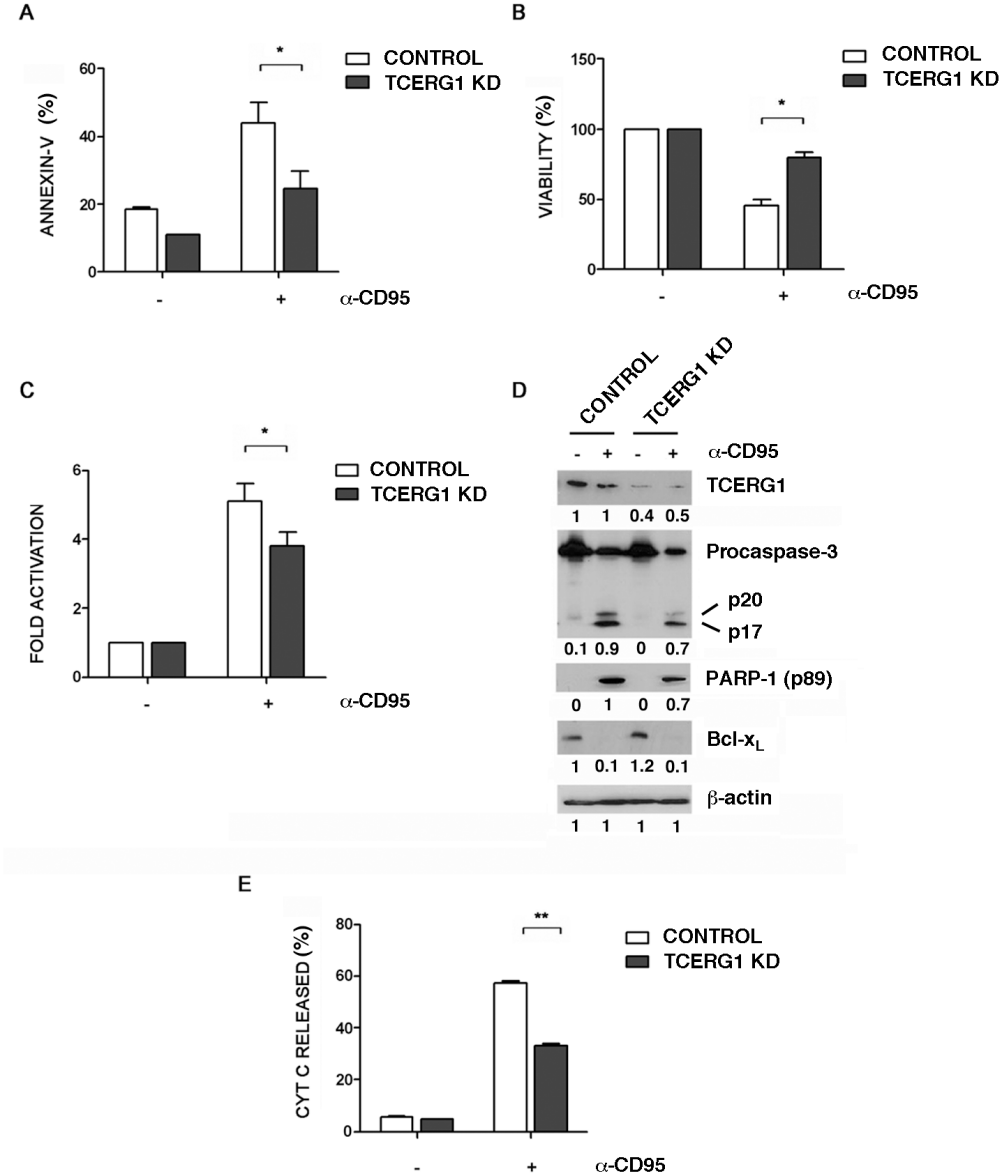
Effect of TCERG1 knockdown on Fas-mediated apoptosis. (A) TCERG1 knockdown reduces annexin-V binding after the induction of Fas-mediated apoptosis. Control and TCERG1 knockdown (KD) Jurkat cells were incubated with annexin-V and analyzed by flow cytometry in the absence (-) or presence (+) of 50 ng/ml of anti-Fas/CD95 for 6 h. The bar graph shows the percentage of annexin-V positive cells from three independent experiments (means ± SEM). *, *p* < 0.05. (B) TCERG1 knockdown recovers the loss in cell viability induced by anti-Fas/CD95 treatment. Control and TCERG1 knockdown (KD) Jurkat cells were incubated in the absence (-) or presence (+) of 50 ng/ml of anti-Fas/CD95 for 6 h. The percentage of viable cells was measured by a luminometric assay. The bar graph shows data from four independent experiments (means ± SEM). *, *p* < 0.05. (C) The activation of caspase-3 is impaired in TCERG1 knockdown Jurkat cells after induction of the extrinsic apoptosis pathway. Caspase-3 activation was measured by chemiluminescence in control and TCERG1 knockdown (KD) Jurkat cells treated with (+) or without (-) α-Fas/CD95 at 50 ng/ml for 6 h. The bar diagram shows the fold activation from four independent experiments (means ± SEM). Data obtained from cells without α-Fas/CD95 treatment were set as 1. *, *p* < 0.05. (D) Analysis of TCERG1, PARP-1 cleavage (p89), procaspase-3 cleavage (precursor p35 and cleaved fragments p20/p17), and Bcl-x_L_ protein expression levels. Protein expression was analyzed by immunoblotting using specific antibodies in protein extracts obtained from control and TCERG1 knockdown Jurkat cells from the experiment described in panel C. ß-actin was used as a loading control. (E) TCERG1 knockdown decreases cytochrome c release from the mitochondria after Fas-mediated apoptosis induction. Control and TCERG1 knockdown (KD) Jurkat cells were incubated in the absence (-) or presence (+) of 50 ng/ml of α-Fas/CD95 for 6 h, and the cytochrome c released was measured using specific antibodies by flow cytometry. The bar graph shows data from two independent experiments and represents the percentage of cytochrome translocated to the cytosol. **, *p* < 0.01.

Apoptosis can also be triggered in a cell through the extrinsic or death receptor-mediated pathway, which is initiated through the stimulation of trans-membrane cell receptors, such as the Fas receptors, located on the cell membrane. To test whether TCERG1 affects the extrinsic apoptosis pathway, we used the agonistic monoclonal anti-Fas/CD95 antibody to induce apoptosis. The anti-Fas/CD95 antibody binds to the membrane receptor Fas (APO-1/CD95), mimicking the endogenous interaction of the receptor with its Fas ligand (FasL) to induce programmed cell death. Annexin-V binding assays revealed that after anti-Fas/CD95 treatment, the control cells showed a higher apoptosis level compared to the TCERG1-knocked down cells (Fig 7A). Under these conditions, we observed an increase in cell viability (Fig 7B) and a decrease in caspase-3 activity (Fig 7C) upon TCERG1 depletion. The analysis of caspase-3 and PARP-1 cleavage by western blotting revealed that the expression level of both apoptotic markers was significantly reduced in the TCERG1-knocked down cells upon apoptosis induction by the anti-Fas/CD95 treatment (Fig 7D). However, no differences were observed in the level of Bcl-x_L_ protein between control and TCERG1 knockdown cells (Fig 7D), suggesting that the effect of TCERG1 with regard to inhibiting Fas-mediated apoptosis does not occur through the regulation of *Bcl-x* alternative splicing. To confirm the role of TCERG1 in the extrinsic apoptosis pathway, we assessed the ΔΨm by measuring mitochondrial cytochrome c release into the cytoplasm upon apoptosis. In agreement with our previous data, we observed significantly reduced cytochrome c release in TCERG1 knockdown cells upon apoptosis induction using the anti-Fas/CD95 antibody (Fig 7E). These data confirm that TCERG1 is involved in the extrinsic apoptosis pathway triggered by the binding of the agonist anti-Fas/CDK9 antibody to the Fas receptor.

### TCERG1 knockdown favors exon 6 skipping of Fas/CD95 mRNA

**Fig 8:**
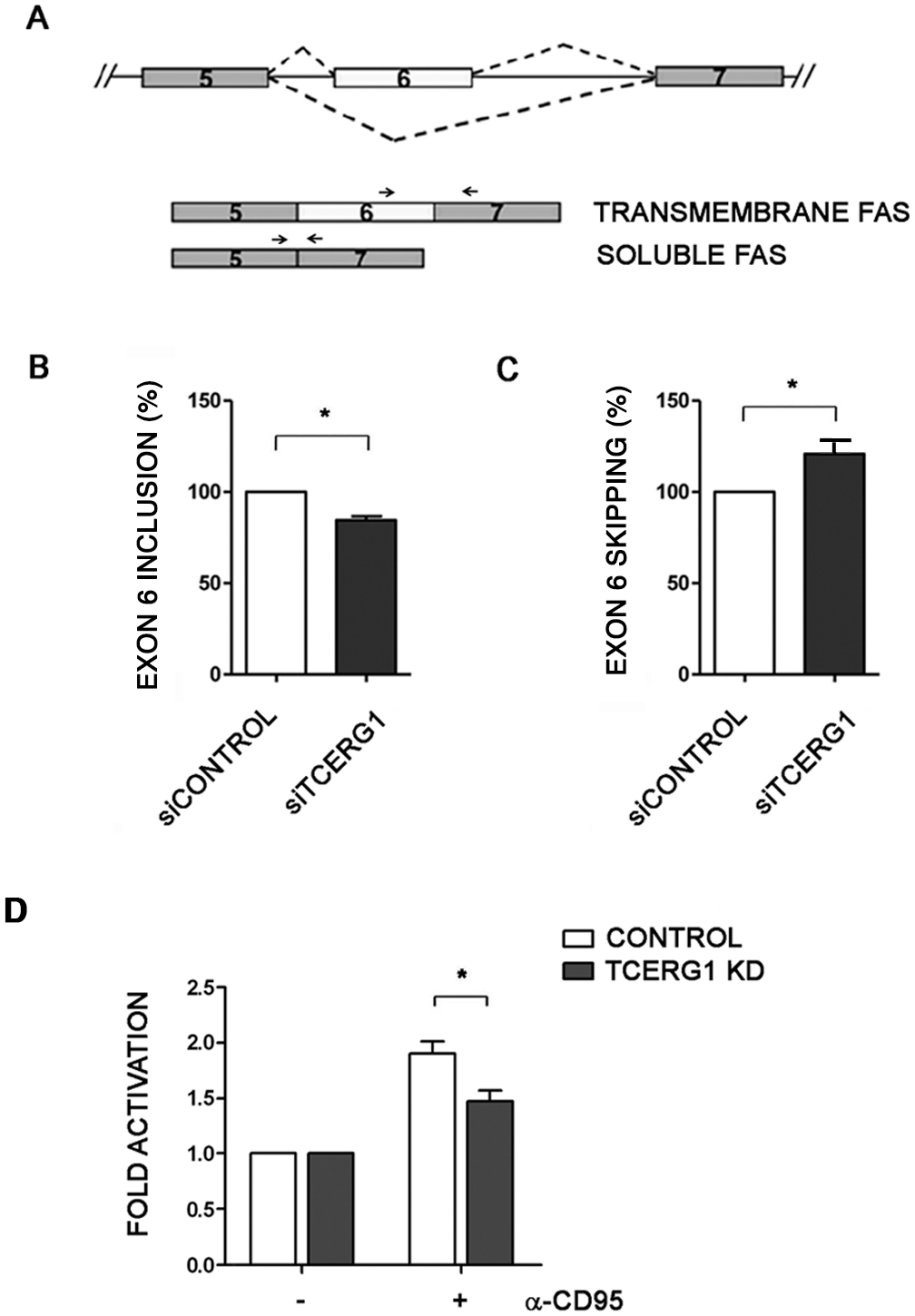
Regulation of *Fas* alternative splicing by TCERG1. (A) Schematic representation of the structure of the *Fas* gene, with exons 5, 6, and 7 (boxes), introns (lines), and alternative splicing events (dotted lines). The alternative Fas isoforms generated by the inclusion or skipping of exon 6 are indicated. The primers used for conventional PCR are shown with black lines and for qPCR as black arrows (B) Analysis of endogenous *Fas* alternative splicing in control and siTCERG1 HEK293T cells. After total RNA extraction, RT-qPCR was carried out using the primers indicated in the panel (black arrows) to amplify the long transmembrane Fas isoform. The bar graph represents the percentage of exon 6 inclusion (means ± SEM). Data are from four independent experiments. *, *p* < 0.05. (C) The same experimental procedures described in B were performed using specific primers to amplify the short soluble Fas isoform. Data are from three independent experiments. *, *p* < 0.05. (D) Analysis of caspase-8 activation in control and TCERG1 knockdown Jurkat cells incubated without (-) or with (+) 50 ng/ml of α-Fas/CD95 for 6 h. Caspase-8 activity was measured by a chemiluminescence assay. The bar graph represents the fold induction of caspase-8 activity under these conditions (means ± SEM). Data are from three independent experiments. *, *p* < 0.05.

The above-mentioned results suggest that the mechanism by which TCERG1 depletion reduces extrinsic apoptosis induction is different than that observed for the intrinsic pathway. Many genes involved in the death-receptor apoptotic pathway are regulated through alternative splicing [3]. One example is the human *Fas* gene. *Fas* mRNA transcripts lacking exon 6 generate a soluble receptor, which can inhibit Fas-mediated apoptosis [12] (Fig 8A). To study the function of TCERG1 in the regulation of *Fas* alternative splicing, we analyzed the splicing pattern of the endogenous *Fas* gene by semiquantitative RT-PCR upon TCERG1 knockdown. Under these conditions, we observed a weak increase in exon 6 skipping (data not shown). To confirm this result, we repeated the analysis by qPCR using primers that exclusively amplify the trans-membrane receptor isoform. Consistent with the previous result, we observed that the absence of TCERG1 promoted a slight but significant decrease of approximately 15–20% in the pro-apoptotic isoform (Fig 8B). To show that more Fas transcript lacking exon 6 is produced after TCERG1 knockdown, we performed the qPCR using primers that exclusively amplify the short transcript isoform. We observed a significant increase in the short anti-apoptotic isoform of approximately 25%, which corroborates the previous data using primers that exclusively amplify the long pro-apoptotic isoform (Fig 8C). We conclude that TCERG1 regulates *Fas* alternative splicing possibly through a mechanism involving exon definition. These results suggest the possibility that TCERG1 regulates apoptosis through the extrinsic pathway by modulating the *Fas* alternative splicing and therefore promoting the accumulation of a specific Fas isoform. To test this hypothesis, we evaluated an event in the apoptosis cascade generated immediately after Fas signaling induction: caspase-8 activation. Caspase-8 is an initiator caspase activated from pro-caspase 8 by the death-inducing signaling complex (DISC) after FasL/Fas interaction [59]. We observed that depletion of TCERG1 caused a significant and reproducible decrease in caspase-8 activation after the induction of apoptosis with the anti-Fas/CD95 antibody (Fig 8D), which is similar to the approximately 20% induction of the pro-apoptotic isoform observed in the splicing experiments (Fig 8B). Taken together, the results indicate than the effect of TCERG1 on extrinsic apoptosis is due, at least partially, to its effect on the regulation of *Fas* alternative splicing.

## Discussion

In this manuscript, we report that TCERG1-mediated *Bcl-x* alternative splicing affects cell apoptosis. Our findings provide a functional link between the control of alternative splicing and the molecular events leading to apoptosis.

The protein kinase inhibitor staurosporine induces a switch in the production of Bcl-x toward the Bcl-x_S_ isoform [28]. The Bcl-x_S_ variant is a pro-apoptotic protein that antagonizes the survival functions of Bcl-x_L_ [60] and sensitizes cells to the apoptosis induced by several agents [19]. Here, we provide evidence that a decrease in the intracellular level of TCERG1 affects the alternative splicing of *Bcl-x* toward the production of the anti-apoptotic Bcl-x_L_ variant after staurosporine treatment. Using multiple experimental approaches, we showed that the splicing activity of TCERG1 affected the induction of apoptosis. We also showed that up-regulation of Bcl-x_S_ expression increased sensitivity to the apoptotic stimulus and counteracted the effect of TCERG1 depletion. Overall, our results indicate the ability of TCERG1 to alter the splice site selection of a key apoptotic gene to regulate cell survival.

Staurosporine is an inhibitor of the PKC pathway that contributes to *Bcl-x* splicing regulation through the SB1 regulatory element [28]. It has been postulated that a splicing repressor present at a nearly limiting concentration binds to SB1 and down-regulates the production of Bcl-x_S_. Thus, blocking the phosphorylation of this putative repressor would suppress its activity and favor degradation of the proteasome-mediated repressor [51]. By stimulating the transcriptional elongation of the *Bcl-x* gene, TCERG1 could reduce the time available for the assembly and activity of this repressor at SB1, thus favoring the production of the Bcl-x_S_ isoform [35]. If this mechanistic scenario is correct, it is possible that only the apoptotic inducers affecting the activity of this splicing repressor would act in the TCERG1-mediated control of apoptosis. The anticancer drug oxaliplatin favors the pro-apoptotic Bcl-x_S_ variant through the same SB1 element that receives signals from the PKC pathway, suggesting that the PKC pathway and the DNA damage signaling route induced by oxaliplatin converge [50, 51]. Our results showed reduced cell death and a higher ΔΨm upon oxaliplatin treatment in Jurkat-shTCERG1 cells, whereas doxorubicin, which promotes apoptosis through a different route, showed no effects on these cells. These data support the idea of staurosporine-dependent effects and suggest a molecular scenario in which the activity of the SB1-binding repressor is being modified.

Our results also suggest a precise mechanism by which TCERG1 modulates the intrinsic apoptosis pathway. Bak is a member of the Bcl-2 protein family that controls apoptosis by regulating the permeability of the mitochondrial outer membrane and release of cytochrome *c* [61]. In healthy cells, Bak is blocked by several anti-apoptotic proteins, such as Bcl-x. Upon induction of apoptosis, Bak undergoes a conformational change that results in the exposure of the amino-terminal region, the release of the anti-apoptotic related proteins, and the formation of homo-oligomers. Our data suggest that upon staurosporine induction, the partial recovery of the Bcl-x_L_ level after TCERG1 knockdown increases the heterodimerization of Bak and Bcl-x_L,_ thus reducing the availability of the amino-terminus of Bak and preventing the initial step of cytochrome *c* release under Bak-mediated mitochondrial apoptosis. Therefore, our data are compatible with a model in which cell death is suppressed by the heterodimerization of Bak and Bcl-x_L_ and in which the dissociation of these proteins leads to mitochondrial permeabilization and the release of proteins that participate in the execution of apoptosis. The precise mechanism of TCERG1 action and the role of other Bcl-2 family members remain unknown.

TCERG1 also affects the extrinsic apoptotic pathway. The down-regulation of TCERG1 decreases the apoptosis induced by anti-Fas/CD95 treatment, though no differences were observed in the Bcl-x_L_ expression level. However, the depletion of TCERG1 provokes a decrease in the pro-apoptotic Fas isoform that correlates with the decreased activity of caspase-8, which is known to be the first step in the cascade of apoptosis events induced by Fas stimulation. The effect in caspase-8 activation does not appear to be sufficient to explain the inhibition of apoptosis observed in cells in which TCERG1 expression is reduced, indicating that later events in the apoptosis cascade might be affected. We cannot discount that other genes implicated in the death receptor pathway that are regulated by alternative splicing might be regulated by TCERG1. For example, Bid, a member of the Bcl-2 family that is activated in response to activation of cell surface death receptors [62], can be expressed in different isoforms that can induce or inhibit apoptosis [63]. It would be interesting to determine whether TCERG1 regulates the alternative splicing of Bid. In support of our data, TCERG1 has been recently identified as a regulator in Fas/CD95 alternative splicing using an automatized genome-wide siRNA screening [64].

We have previously reported that TCERG1 could modulate the elongation rate of RNAPII to relieve a putative polymerase pause site for proper pre-mRNA synthesis and processing [35]. Recent studies suggest that pausing could act as a checkpoint to ensure the optimal activity of RNAPII during development, especially in highly regulated or inducible genes [65, 66]. This checkpoint could serve as a tool to unify responses to different signaling pathways or stimuli. The alternative splicing of multiple genes is altered in response to signaling pathways [67]. In the case of the alternative splicing of *Bcl-x*, apoptosis is regulated by signals that include the *de novo* synthesis of ceramide and phosphatase PP1 [31]. PKC inhibitors also alter *Bcl-x* alternative splicing in healthy but not in oncogenic cells [28], and a signaling pathway through PI3K is responsible for the changes in the Bcl-x_L_/Bcl-x_S_ mRNA ratio in cancer tissues by affecting the expression of the splicing factor SAP155 [68]. These data indicate that specific signaling pathways regulate the alternative splicing of important apoptosis genes. TCER-1, the *Caenorhabditis elegans* TCERG1 homolog, responds to reproductive signals and promotes the expression of a set of genes regulated by the conserved, life-extending transcription factor DAF-16/FOXO [69]. An attractive possibility is that TCERG1 acts as a checkpoint in response to different stimuli, such as apoptosis induction, to control transcription elongation, alternative splicing, or other post-transcriptional steps [70].

The physiological relevance of the Bcl-x_L_/Bcl-x_S_ mRNA ratio has been demonstrated in multiple studies [32, 68, 71, 72]. In addition, many cancer cells overexpress Bcl-x_L_, and this isoform cooperates with Myc to promote oncogenic transformation by blocking Myc-induced apoptosis [73]. Fas receptor ratios are also important for development or disease, and the differential expression of alternatively spliced Fas mRNA isoforms is associated with lymphocyte activation, autoimmune lymphoproliferative syndrome, systemic lupus erythematosus, and systemic sclerosis [74–77]. Hence, manipulation of the ratio of alternatively spliced mRNA isoforms of genes is a promising strategy to sensitize the cells to therapeutic agents. The results described in the present study suggest that the regulation of the alternative splicing of the *Bcl*-x and *Fas* apoptotic genes by TCERG1 is a relevant regulatory pathway in modulating apoptotic responses to sensitize a cell to apoptotic inductors.

## References

[1] Pan Q, Shai O, Lee LJ, Frey BJ, Blencowe BJ. Deep surveying of alternative splicing complexity in the human transcriptome by high-throughput sequencing. Nat Genet. 2008;40(12):1413–5. Epub 2008/11/04. ng.259 [pii] 10.1038/ng.259 . 18978789

[2] Wang ET, Sandberg R, Luo S, Khrebtukova I, Zhang L, Mayr C, et al Alternative isoform regulation in human tissue transcriptomes. Nature. 2008;456(7221):470–6. Epub 2008/11/04. 10.1038/nature07509 18978772 PMC2593745

[3] Schwerk C, Schulze-Osthoff K. Regulation of apoptosis by alternative pre-mRNA splicing. Mol Cell. 2005;19(1):1–13. Epub 2005/07/02. 10.1016/j.molcel.2005.05.026 . 15989960

[4] Ule J, Darnell RB. Functional and mechanistic insights from genome-wide studies of splicing regulation in the brain. Adv Exp Med Biol. 2007;623:148–60. Epub 2008/04/03. . 18380345 10.1007/978-0-387-77374-2_9

[5] Biamonti G, Caceres JF. Cellular stress and RNA splicing. Trends Biochem Sci. 2009;34(3):146–53. Epub 2009/02/12. 10.1016/j.tibs.2008.11.004 . 19208481

[6] Wang GS, Cooper TA. Splicing in disease: disruption of the splicing code and the decoding machinery. Nat Rev Genet. 2007;8(10):749–61. Epub 2007/08/30. nrg2164 [pii] 10.1038/nrg2164 . 17726481

[7] Cotter TG, Lennon SV, Glynn JG, Martin SJ. Cell death via apoptosis and its relationship to growth, development and differentiation of both tumour and normal cells. Anticancer research. 1990;10(5A):1153–9. Epub 1990/09/01. . 2241096

[8] McKenna SL, McGowan AJ, Cotter TG. Molecular mechanisms of programmed cell death. Advances in biochemical engineering/biotechnology. 1998;62:1–31. Epub 1998/10/02. 9755639 10.1007/BFb0102304

[9] Dowling P, Shang G, Raval S, Menonna J, Cook S, Husar W. Involvement of the CD95 (APO-1/Fas) receptor/ligand system in multiple sclerosis brain. The Journal of experimental medicine. 1996;184(4):1513–8. Epub 1996/10/01. 8879222 10.1084/jem.184.4.1513 PMC2192814

[10] Singh S, Dikshit M. Apoptotic neuronal death in Parkinson's disease: involvement of nitric oxide. Brain research reviews. 2007;54(2):233–50. Epub 2007/04/06. 10.1016/j.brainresrev.2007.02.001 . 17408564

[11] Fuchs Y, Steller H. Programmed cell death in animal development and disease. Cell. 2011;147(4):742–58. Epub 2011/11/15. 10.1016/j.cell.2011.10.033 . 22078876 PMC4511103

[12] Cheng J, Zhou T, Liu C, Shapiro JP, Brauer MJ, Kiefer MC, et al Protection from Fas-mediated apoptosis by a soluble form of the Fas molecule. Science. 1994;263(5154):1759–62. Epub 1994/03/25. . 7510905 10.1126/science.7510905

[13] Cascino I, Fiucci G, Papoff G, Ruberti G. Three functional soluble forms of the human apoptosis-inducing Fas molecule are produced by alternative splicing. J Immunol. 1995;154(6):2706–13. Epub 1995/03/15. . 7533181

[14] Akgul C, Moulding DA, Edwards SW. Alternative splicing of Bcl-2-related genes: functional consequences and potential therapeutic applications. Cell Mol Life Sci. 2004;61(17):2189–99. Epub 2004/09/01. 10.1007/s00018-004-4001-7 . 15338051 PMC11138917

[15] Boise LH, Gonzalez-Garcia M, Postema CE, Ding L, Lindsten T, Turka LA, et al bcl-x, a bcl-2-related gene that functions as a dominant regulator of apoptotic cell death. Cell. 1993;74(4):597–608. Epub 1993/08/27. . 8358789 10.1016/0092-8674(93)90508-n

[16] Olopade OI, Adeyanju MO, Safa AR, Hagos F, Mick R, Thompson CB, et al Overexpression of BCL-x protein in primary breast cancer is associated with high tumor grade and nodal metastases. The cancer journal from Scientific American. 1997;3(4):230–7. Epub 1997/07/01. . 9263629

[17] Krajewski S, Krajewska M, Shabaik A, Wang HG, Irie S, Fong L, et al Immunohistochemical analysis of in vivo patterns of Bcl-X expression. Cancer Res. 1994;54(21):5501–7. Epub 1994/11/01. . 7923184

[18] Clarke MF, Apel IJ, Benedict MA, Eipers PG, Sumantran V, Gonzalez-Garcia M, et al A recombinant bcl-x s adenovirus selectively induces apoptosis in cancer cells but not in normal bone marrow cells. Proc Natl Acad Sci U S A. 1995;92(24):11024–8. Epub 1995/11/21. 7479929 10.1073/pnas.92.24.11024 PMC40563

[19] Sumantran VN, Ealovega MW, Nunez G, Clarke MF, Wicha MS. Overexpression of Bcl-XS sensitizes MCF-7 cells to chemotherapy-induced apoptosis. Cancer Res. 1995;55(12):2507–10. Epub 1995/06/15. . 7780958

[20] Lindenboim L, Yuan J, Stein R. Bcl-xS and Bax induce different apoptotic pathways in PC12 cells. Oncogene. 2000;19(14):1783–93. Epub 2000/04/25. 10.1038/sj.onc.1203495 . 10777212

[21] Leu S, Lin YM, Wu CH, Ouyang P. Loss of Pnn expression results in mouse early embryonic lethality and cellular apoptosis through SRSF1-mediated alternative expression of Bcl-xS and ICAD. J Cell Sci. 2012;125(Pt 13):3164–72. Epub 2012/03/29. 10.1242/jcs.100859 . 22454513

[22] Kim R. Unknotting the roles of Bcl-2 and Bcl-xL in cell death. Biochem Biophys Res Commun. 2005;333(2):336–43. Epub 2005/06/01. 10.1016/j.bbrc.2005.04.161 . 15922292

[23] Villunger A, Labi V, Bouillet P, Adams J, Strasser A. Can the analysis of BH3-only protein knockout mice clarify the issue of 'direct versus indirect' activation of Bax and Bak? Cell death and differentiation. 2011;18(10):1545–6. Epub 2011/09/13. 10.1038/cdd.2011.100 21909118 PMC3172109

[24] Degli Esposti M, Dive C. Mitochondrial membrane permeabilisation by Bax/Bak. Biochem Biophys Res Commun. 2003;304(3):455–61. Epub 2003/05/06. . 12729579 10.1016/s0006-291x(03)00617-x

[25] Garneau D, Revil T, Fisette JF, Chabot B. Heterogeneous nuclear ribonucleoprotein F/H proteins modulate the alternative splicing of the apoptotic mediator Bcl-x. J Biol Chem. 2005;280(24):22641–50. Epub 2005/04/20. 10.1074/jbc.M501070200 . 15837790

[26] Paradis C, Cloutier P, Shkreta L, Toutant J, Klarskov K, Chabot B. hnRNP I/PTB can antagonize the splicing repressor activity of SRp30c. RNA. 2007;13(8):1287–300. Epub 2007/06/06. 10.1261/rna.403607 17548433 PMC1924885

[27] Paronetto MP, Achsel T, Massiello A, Chalfant CE, Sette C. The RNA-binding protein Sam68 modulates the alternative splicing of Bcl-x. J Cell Biol. 2007;176(7):929–39. Epub 2007/03/21. 10.1083/jcb.200701005 17371836 PMC2064079

[28] Revil T, Toutant J, Shkreta L, Garneau D, Cloutier P, Chabot B. Protein kinase C-dependent control of Bcl-x alternative splicing. Mol Cell Biol. 2007;27(24):8431–41. Epub 2007/10/10. MCB.00565-07 [pii] 10.1128/MCB.00565-07 17923691 PMC2169420

[29] Cloutier P, Toutant J, Shkreta L, Goekjian S, Revil T, Chabot B. Antagonistic effects of the SRp30c protein and cryptic 5' splice sites on the alternative splicing of the apoptotic regulator Bcl-x. J Biol Chem. 2008;283(31):21315–24. Epub 2008/06/07. 10.1074/jbc.M800353200 . 18534987

[30] Chalfant CE, Ogretmen B, Galadari S, Kroesen BJ, Pettus BJ, Hannun YA. FAS activation induces dephosphorylation of SR proteins; dependence on the de novo generation of ceramide and activation of protein phosphatase 1. J Biol Chem. 2001;276(48):44848–55. Epub 2001/08/15. 10.1074/jbc.M106291200 . 11502750

[31] Chalfant CE, Rathman K, Pinkerman RL, Wood RE, Obeid LM, Ogretmen B, et al De novo ceramide regulates the alternative splicing of caspase 9 and Bcl-x in A549 lung adenocarcinoma cells. Dependence on protein phosphatase-1. J Biol Chem. 2002;277(15):12587–95. Epub 2002/01/22. 10.1074/jbc.M112010200 . 11801602

[32] Taylor JK, Zhang QQ, Wyatt JR, Dean NM. Induction of endogenous Bcl-xS through the control of Bcl-x pre-mRNA splicing by antisense oligonucleotides. Nat Biotechnol. 1999;17(11):1097–100. Epub 1999/11/05. 10.1038/15079 . 10545916

[33] Kim K. Silencing Bcl-X(L) in cancer therapy. Cancer biology & therapy. 2005;4(4):398–9. Epub 2005/05/24. 15908790 10.4161/cbt.4.4.1761

[34] Adams JM, Cory S. The Bcl-2 apoptotic switch in cancer development and therapy. Oncogene. 2007;26(9):1324–37. Epub 2007/02/27. 10.1038/sj.onc.1210220 17322918 PMC2930981

[35] Montes M, Cloutier A, Sánchez-Hernández N, Michelle L, Lemieux B, Blanchette M, et al TCERG1 regulates alternative splicing of Bcl-x gene by modulating the rate of RNAPII transcription. Mol Cell Biol. 2012;32:751–62. Epub 2011/12/14. MCB.06255-11 [pii]. 22158966 10.1128/MCB.06255-11 PMC3272968

[36] Wang KC, Cheng AL, Chuang SE, Hsu HC, Su IJ. Retinoic acid-induced apoptotic pathway in T-cell lymphoma: Identification of four groups of genes with differential biological functions. Exp Hematol. 2000;28(12):1441–50. Epub 2001/01/09. S0301-472X(00)00546-4 [pii]. . 11146166 10.1016/s0301-472x(00)00546-4

[37] Smith MJ, Kulkarni S, Pawson T. FF domains of CA150 bind transcription and splicing factors through multiple weak interactions. Mol Cell Biol. 2004;24(21):9274–85. . 15485897 10.1128/MCB.24.21.9274-9285.2004 PMC522232

[38] Suñé C, Hayashi T, Liu Y, Lane WS, Young RA, Garcia-Blanco MA. CA150, a nuclear protein associated with the RNA polymerase II holoenzyme, is involved in Tat-activated human immunodeficiency virus type 1 transcription. Mol Cell Biol. 1997;17(10):6029–39. . 9315662 10.1128/mcb.17.10.6029 PMC232452

[39] Goldstrohm AC, Albrecht TR, Suñé C, Bedford MT, Garcia-Blanco MA. The transcription elongation factor CA150 interacts with RNA polymerase II and the pre-mRNA splicing factor SF1. Mol Cell Biol. 2001;21(22):7617–28. Epub 2001/10/18. PubMed Central PMCID: PMC99933. 11604498 10.1128/MCB.21.22.7617-7628.2001 PMC99933

[40] Lin KT, Lu RM, Tarn WY. The WW domain-containing proteins interact with the early spliceosome and participate in pre-mRNA splicing in vivo. Mol Cell Biol. 2004;24(20):9176–85. Epub 2004/10/01. PubMed Central PMCID: PMC517884. 15456888 10.1128/MCB.24.20.9176-9185.2004 PMC517884

[41] Sánchez-Álvarez M, Goldstrohm AC, Garcia-Blanco MA, Suñé C. Human transcription elongation factor CA150 localizes to splicing factor-rich nuclear speckles and assembles transcription and splicing components into complexes through its amino and carboxyl regions. Mol Cell Biol. 2006;26(13):4998–5014. Epub 2006/06/20. 26/13/4998 [pii] 10.1128/MCB.01991-05 16782886 PMC1489151

[42] Cheng D, Cote J, Shaaban S, Bedford MT. The arginine methyltransferase CARM1 regulates the coupling of transcription and mRNA processing. Mol Cell. 2007;25(1):71–83. . 17218272 10.1016/j.molcel.2006.11.019

[43] Pearson JL, Robinson TJ, Munoz MJ, Kornblihtt AR, Garcia-Blanco MA. Identification of the cellular targets of the transcription factor TCERG1 reveals a prevalent role in mRNA processing. J Biol Chem. 2008;283(12):7949–61. Epub 2008/01/12. M709402200 [pii] 10.1074/jbc.M709402200 . 18187414

[44] Sánchez-Álvarez M, Montes M, Sánchez-Hernández N, Hernández-Munain C, Suñé C. Differential effects of sumoylation on transcription and alternative splicing by transcription elongation regulator 1 (TCERG1). J Biol Chem. 2010;285(20):15220–33. Epub 2010/03/11. M109.063750 [pii] 10.1074/jbc.M109.063750 20215116 PMC2865347

[45] Montes M, Becerra S, Sanchez-Alvarez M, Sune C. Functional coupling of transcription and splicing. Gene. 2012;501(2):104–17. Epub 2012/04/28. 10.1016/j.gene.2012.04.006 . 22537677

[46] Coiras M, Montes M, Montanuy I, Lopez-Huertas MR, Mateos E, Le Sommer C, et al Transcription elongation regulator 1 (TCERG1) regulates competent RNA polymerase II-mediated elongation of HIV-1 transcription and facilitates efficient viral replication. Retrovirology. 2013;10(1):124 Epub 2013/10/30. 10.1186/1742-4690-10-124 24165037 PMC3874760

[47] Suñé C, Garcia-Blanco MA. Transcriptional cofactor CA150 regulates RNA polymerase II elongation in a TATA-box-dependent manner. Mol Cell Biol. 1999;19(7):4719–28. . 10373521 10.1128/mcb.19.7.4719 PMC84270

[48] Lopez-Huertas MR, Callejas S, Abia D, Mateos E, Dopazo A, Alcami J, et al Modifications in host cell cytoskeleton structure and function mediated by intracellular HIV-1 Tat protein are greatly dependent on the second coding exon. Nucleic Acids Res. 2010;38(10):3287–307. Epub 2010/02/09. 10.1093/nar/gkq037 20139419 PMC2879518

[49] Gewirtz DA. A critical evaluation of the mechanisms of action proposed for the antitumor effects of the anthracycline antibiotics adriamycin and daunorubicin. Biochemical pharmacology. 1999;57(7):727–41. Epub 1999/03/13. . 10075079 10.1016/s0006-2952(98)00307-4

[50] Shkreta L, Froehlich U, Paquet ER, Toutant J, Elela SA, Chabot B. Anticancer drugs affect the alternative splicing of Bcl-x and other human apoptotic genes. Molecular cancer therapeutics. 2008;7(6):1398–409. Epub 2008/06/21. 10.1158/1535-7163.MCT-08-0192 . 18566212

[51] Shkreta L, Michelle L, Toutant J, Tremblay ML, Chabot B. The DNA damage response pathway regulates the alternative splicing of the apoptotic mediator Bcl-x. J Biol Chem. 2011;286(1):331–40. Epub 2010/10/29. M110.162644 [pii] 10.1074/jbc.M110.162644 20980256 PMC3012990

[52] Griffiths GJ, Dubrez L, Morgan CP, Jones NA, Whitehouse J, Corfe BM, et al Cell damage-induced conformational changes of the pro-apoptotic protein Bak in vivo precede the onset of apoptosis. J Cell Biol. 1999;144(5):903–14. Epub 1999/03/23. 10085290 10.1083/jcb.144.5.903 PMC2148192

[53] Cheng EH, Levine B, Boise LH, Thompson CB, Hardwick JM. Bax-independent inhibition of apoptosis by Bcl-XL. Nature. 1996;379(6565):554–6. Epub 1996/02/08. 10.1038/379554a0 . 8596636

[54] Chittenden T, Flemington C, Houghton AB, Ebb RG, Gallo GJ, Elangovan B, et al A conserved domain in Bak, distinct from BH1 and BH2, mediates cell death and protein binding functions. EMBO J. 1995;14(22):5589–96. Epub 1995/11/15. 8521816 10.1002/j.1460-2075.1995.tb00246.x PMC394673

[55] Farrow SN, White JH, Martinou I, Raven T, Pun KT, Grinham CJ, et al Cloning of a bcl-2 homologue by interaction with adenovirus E1B 19K. Nature. 1995;374(6524):731–3. Epub 1995/04/20. 10.1038/374731a0 . 7715729

[56] Simonian PL, Grillot DA, Nunez G. Bak can accelerate chemotherapy-induced cell death independently of its heterodimerization with Bcl-XL and Bcl-2. Oncogene. 1997;15(15):1871–5. Epub 1997/11/15. 10.1038/sj.onc.1201350 . 9362454

[57] Vaux DL. Apoptogenic factors released from mitochondria. Biochimica et biophysica acta. 2011;1813(4):546–50. Epub 2010/08/18. 10.1016/j.bbamcr.2010.08.002 . 20713095

[58] Cheng EH, Wei MC, Weiler S, Flavell RA, Mak TW, Lindsten T, et al BCL-2, BCL-X(L) sequester BH3 domain-only molecules preventing BAX- and BAK-mediated mitochondrial apoptosis. Mol Cell. 2001;8(3):705–11. Epub 2001/10/05. . 11583631 10.1016/s1097-2765(01)00320-3

[59] Kischkel FC, Hellbardt S, Behrmann I, Germer M, Pawlita M, Krammer PH, et al Cytotoxicity-dependent APO-1 (Fas/CD95)-associated proteins form a death-inducing signaling complex (DISC) with the receptor. EMBO J. 1995;14(22):5579–88. Epub 1995/11/15. 8521815 10.1002/j.1460-2075.1995.tb00245.x PMC394672

[60] Minn AJ, Boise LH, Thompson CB. Bcl-x(S) anatagonizes the protective effects of Bcl-x(L). J Biol Chem. 1996;271(11):6306–12. Epub 1996/03/15. . 8626425 10.1074/jbc.271.11.6306

[61] Shamas-Din A, Brahmbhatt H, Leber B, Andrews DW. BH3-only proteins: Orchestrators of apoptosis. Biochimica et biophysica acta. 2011;1813(4):508–20. Epub 2010/12/15. 10.1016/j.bbamcr.2010.11.024 . 21146563

[62] Luo X, Budihardjo I, Zou H, Slaughter C, Wang X. Bid, a Bcl2 interacting protein, mediates cytochrome c release from mitochondria in response to activation of cell surface death receptors. Cell. 1998;94(4):481–90. Epub 1998/09/04. . 9727491 10.1016/s0092-8674(00)81589-5

[63] Renshaw SA, Dempsey CE, Barnes FA, Bagstaff SM, Dower SK, Bingle CD, et al Three novel Bid proteins generated by alternative splicing of the human Bid gene. J Biol Chem. 2004;279(4):2846–55. Epub 2003/10/30. 10.1074/jbc.M309769200 . 14583606

[64] Tejedor JR, Papasaikas P, Valcarcel J. Genome-wide identification of Fas/CD95 alternative splicing regulators reveals links with iron homeostasis. Mol Cell. 2015;57(1):23–38. Epub 2014/12/09. 10.1016/j.molcel.2014.10.029 . 25482508

[65] Gilchrist DA, Dos Santos G, Fargo DC, Xie B, Gao Y, Li L, et al Pausing of RNA polymerase II disrupts DNA-specified nucleosome organization to enable precise gene regulation. Cell. 2010;143(4):540–51. Epub 2010/11/16. S0092-8674(10)01140-2 [pii] 10.1016/j.cell.2010.10.004 21074046 PMC2991113

[66] Levine M. Paused RNA Polymerase II as a Developmental Checkpoint. Cell. 2011;145(4):502–11. Epub 2011/05/14. S0092-8674(11)00479-X [pii] 10.1016/j.cell.2011.04.021 . 21565610 PMC4257488

[67] Shin C, Manley JL. Cell signalling and the control of pre-mRNA splicing. Nat Rev Mol Cell Biol. 2004;5(9):727–38. Epub 2004/09/02. 10.1038/nrm1467 . 15340380

[68] Shultz JC, Vu N, Shultz MD, Mba MU, Shapiro BA, Chalfant CE. The Proto-oncogene PKCiota regulates the alternative splicing of Bcl-x pre-mRNA. Molecular cancer research: MCR. 2012;10(5):660–9. Epub 2012/04/24. 10.1158/1541-7786.MCR-11-0363 22522453 PMC3356487

[69] Ghazi A, Henis-Korenblit S, Kenyon C. A transcription elongation factor that links signals from the reproductive system to lifespan extension in Caenorhabditis elegans. PLoS Genet. 2009;5(9):e1000639 Epub 2009/09/15. 10.1371/journal.pgen.1000639 19749979 PMC2729384

[70] Pushpa K, Kumar GA, Subramaniam K. PUF-8 and TCER-1 are essential for normal levels of multiple mRNAs in the C. elegans germline. Development. 2013;140(6):1312–20. Epub 2013/02/28. 10.1242/dev.087833 23444359 PMC3585663

[71] Mercatante DR, Bortner CD, Cidlowski JA, Kole R. Modification of alternative splicing of Bcl-x pre-mRNA in prostate and breast cancer cells. analysis of apoptosis and cell death. J Biol Chem. 2001;276(19):16411–7. Epub 2001/03/30. 10.1074/jbc.M009256200 . 11278482

[72] Mercatante DR, Sazani P, Kole R. Modification of alternative splicing by antisense oligonucleotides as a potential chemotherapy for cancer and other diseases. Current cancer drug targets. 2001;1(3):211–30. Epub 2002/08/22. . 12188880 10.2174/1568009013334124

[73] Finch A, Prescott J, Shchors K, Hunt A, Soucek L, Dansen TB, et al Bcl-xL gain of function and p19 ARF loss of function cooperate oncogenically with Myc in vivo by distinct mechanisms. Cancer cell. 2006;10(2):113–20. Epub 2006/08/15. 10.1016/j.ccr.2006.06.017 . 16904610

[74] Liu C, Cheng J, Mountz JD. Differential expression of human Fas mRNA species upon peripheral blood mononuclear cell activation. Biochem J. 1995;310 (Pt 3):957–63. Epub 1995/09/15. 7575433 10.1042/bj3100957 PMC1135989

[75] Roesler J, Izquierdo JM, Ryser M, Rosen-Wolff A, Gahr M, Valcarcel J, et al Haploinsufficiency, rather than the effect of an excessive production of soluble CD95 (CD95{Delta}TM), is the basis for ALPS Ia in a family with duplicated 3' splice site AG in CD95 intron 5 on one allele. Blood. 2005;106(5):1652–9. Epub 2005/05/05. 10.1182/blood-2004-08-3104 . 15870181

[76] Wetzig T, Petri JB, Mittag M, Haustein UF. Serum levels of soluble Fas/APO-1 receptor are increased in systemic sclerosis. Archives of dermatological research. 1998;290(4):187–90. Epub 1998/06/09. . 9617437 10.1007/s004030050288

[77] Al-Maini MH, Mountz JD, Al-Mohri HA, El-Ageb EM, Al-Riyami BM, Svenson KL, et al Serum levels of soluble Fas correlate with indices of organ damage in systemic lupus erythematosus. Lupus. 2000;9(2):132–9. Epub 2000/04/29. . 10787011 10.1191/096120300678828145

